# Oral versus vaginal *Lactobacillus* supplementation for endometrial dysbiosis: A paired pre and post–endometrial microbiome study

**DOI:** 10.1371/journal.pone.0360619

**Published:** 2026-10-26

**Authors:** Daiki Hiratsuka, Ayumi Iki, Mayu Kinoshita, Yumiko Doi, Okikaze Kato, Kotaro Oshima, Takehiro Hiraoka, Mitsunori Matsuo, Mari Ichinose, Tomoko Makabe, Gentaro Izumi, Miyuki Harada, Yasushi Hirota, Motowo Nabeta

**Affiliations:** 1 Department of Obstetrics and Gynecology, Graduate School of Medicine, The University of Tokyo, Tokyo, Japan; 2 Tsubaki Women’s Clinic, Matsuyama, Ehime, Japan; Universidade dos Açores Departamento de Biologia: Universidade dos Acores Departamento de Biologia, PORTUGAL

## Abstract

Infertility is a global health challenge, and impaired endometrial receptivity remains an important contributor to treatment failure. Endometrial dysbiosis, characterized by a low proportion of *Lactobacillus* in the endometrial microbiota, has been proposed as a potentially modifiable factor that may affect endometrial receptivity and fertility, and treatment with *Lactobacillus*-containing probiotics is emerging as a promising therapeutic approach. However, the optimal route of *Lactobacillus* supplementation remains unclear. We compared oral versus vaginal supplementation using the same *Lactobacillus*-containing formulation as an adjunct to standardized doxycycline/metronidazole therapy and continued oral lactoferrin in patients with baseline endometrial *Lactobacillus* <10%, based on paired microbiome profiling of endometrial samples (aspirated intrauterine fluid and endometrial tissue). In this single-center retrospective cohort study, thirty-two patients underwent paired endometrial microbiome testing before and after 30 days of supplementation (oral, n = 18; vaginal, n = 14). In the vaginal group, the median endometrial *Lactobacillus* proportion increased from 0.05% [0.00–0.20] to 99.15% [70.45–100.00] (p = 0.001), whereas in the oral group it increased from 0.00% [0.00–0.18] to 0.30% [0.03–66.33] (p = 0.005). The median change was greater with vaginal versus oral supplementation (+98.90% [70.45–99.80] vs + 0.25% [0.03–66.33], p < 0.001). Post-treatment *Lactobacillus* ≥80%, used as an exploratory marker of marked microbiota improvement, was observed in 9/14 patients (64%) in the vaginal group and 3/18 (17%) in the oral group (p = 0.010). Exploratory reproductive outcomes were limited by the small sample size and did not define a reliable clinical threshold. These findings suggest that, within this combined treatment regimen, vaginal *Lactobacillus* supplementation may provide a greater route-associated additional improvement in endometrial microbiota profiles than oral administration.

## Introduction

Infertility is a global health concern that affects approximately one in six couples and remains a major challenge in reproductive medicine [1]. Although advances in assisted reproductive technology (ART) have substantially improved the overall success rates, a considerable proportion of patients still fail to achieve pregnancy and live birth despite repeated ART attempts [2]. The causes of ART failure can be broadly categorized into embryonic and uterine factors [3]. Embryo-related implantation failure has been increasingly addressed through advances in preimplantation genetic testing (PGT) [4]. However, therapeutic strategies targeting uterine-related implantation failure remain limited [4–6]. Established interventions are largely confined to the surgical correction of overt intrauterine abnormalities, such as endometrial polyps or uterine fibroids [7–9]. Consequently, implantation failure, which is potentially attributable to impaired endometrial receptivity, continues to represent a major barrier to pregnancy.

In recent years, increasing attention has been directed toward the potential role of endometrial microbiota in reproductive outcomes. The *Lactobacillus*-dominant microbiota (LDM) has been associated with higher implantation success, whereas the non-*Lactobacillus*-dominant microbiota (NLDM) is often regarded as dysbiotic and has been reported more frequently among infertile women [10–13]. Notably, NLDM prevalence in this population is estimated to be approximately 50% [14], highlighting endometrial dysbiosis as a potentially important and modifiable uterine factor contributing to infertility.

In a related condition, bacterial vaginosis, supplementation with probiotics containing live *Lactobacillus* species has been shown to be beneficial for restoring microbial balance [15,16]; however, whether outcomes differ according to vaginal or oral administration remains a matter of debate [17–19]. Similarly, several studies have suggested that *Lactobacillus*-containing products may improve endometrial microbiota profiles in women with endometrial dysbiosis [20–22]. Two reports have further implied that vaginal administration may be associated with a larger increase in endometrial *Lactobacillus* dominance than oral supplementation [23,24]. Although these studies support the potential relevance of *Lactobacillus* supplementation, direct route comparisons in the endometrium remain limited, particularly studies using the same formulation and paired pre- and post-treatment endometrial microbiome assessments [12,13].

Therefore, this study aimed to compare route-associated changes in endometrial *Lactobacillus* abundance after oral versus vaginal *Lactobacillus* supplementation, administered as an adjunct to standardized doxycycline/metronidazole therapy and continued oral lactoferrin, in patients with endometrial dysbiosis who underwent paired pre- and post-supplementation endometrial microbiome testing.

## Materials and methods

### Study design and data collection

This single-center retrospective cohort study with paired pre–post assessments was conducted in accordance with the Declaration of Helsinki and the Ethical Guidelines for Medical and Biological Research Involving Human Subjects established by the Japanese Government. This study was conducted as an institutional collaboration between the University of Tokyo and Tsubaki Women’s Clinic, with centralized ethical review at the University of Tokyo. The study protocol was reviewed and approved by the Research Ethics Committee of the Faculty of Medicine at the University of Tokyo (Institutional Review Board No. 2025340NI) on 13 November 2025. Following this approval, institutional permission was obtained from the Tsubaki Women’s Clinic, where the study was conducted.

Given the retrospective nature of the study, the requirement for written informed consent was waived, and was substituted with an opt-out procedure. The waiver of written consent and use of the opt-out procedure were approved by the Research Ethics Committee of the Faculty of Medicine, University of Tokyo.

Following institutional approval, clinical data were retrospectively extracted from electronic medical records at Tsubaki Women’s Clinic by MN and AI, who anonymized the dataset before analysis. Medical records were accessed for research purposes on 1 February 2026. At Tsubaki Women’s Clinic, limited identifiable information was used solely for record linkage and data extraction; thereafter, the dataset was de-identified prior to analysis. DH performed the statistical analyses using the anonymized dataset. The authors did not have access to information that could identify individual participants during or after the analytical phase. Forty infertile patients who underwent an endometrial microbiome test were screened before any infertility treatment between January 2022 and January 2023, and were found to have a *Lactobacillus* proportion of < 10%. To reduce potential confounding by evident structural pathology, patients with uterine or pelvic organic lesions, including leiomyomas, endometrial polyps, adenomyosis, or endometriosis, were excluded.

Because all eligible patients had a low endometrial *Lactobacillus* proportion before treatment, all patients received standardized concomitant therapy consisting of doxycycline (200 mg on day one, followed by 100 mg once daily from day two for 14 days), metronidazole (250 mg twice daily for 14 days), and continued oral lactoferrin. In addition, all patients received one capsule daily for 30 days via either the vaginal or oral route. Patients who did not undergo a second endometrial microbiome test after supplementation owing to loss to follow-up or clinical pregnancy before the second test were excluded from the paired pre–post microbiome analysis, because endometrial microbiome testing cannot be performed during pregnancy.

The collected clinical data included age, body mass index (BMI), gravidity, parity, prior infertility treatment at another clinic, endometrial CD138 positivity, subsequent clinical pregnancy, and live births. Chronic endometritis (CE) was assessed as a baseline clinical characteristic by CD138 immunostaining and defined as the presence of ≥ 1 CD138-positive plasma cell in 10 high-power fields (HPFs, × 400 magnification) [25]. Pregnancy outcomes were ascertained by medical record review for up to 2 years after the second endometrial microbiome test.

### *Lactobacillus*-containing probiotics used in this study

All participants received the same commercially available probiotic formulation containing *Lacticaseibacillus rhamnosus* HN001™ (6 × 10^9^ colony-forming units [CFU]) and *Lactobacillus acidophilus* LA-14® (9 × 10^9^ CFU) per capsule. The same capsule formulation, without any route-specific modification, was used for both oral and vaginal administration.

According to information provided by the manufacturer, the capsule shell consisted primarily of hydroxypropyl methylcellulose, with small amounts of carrageenan and potassium chloride and trace amounts of dimethylpolysiloxane and sorbitan fatty acid ester. The formulation also contained starch, calcium stearate, and silicon dioxide as excipients, and the capsule had no special enteric or other functional coating. The product met the applicable Japanese Pharmacopoeia disintegration requirements for oral use. In addition, prior to the present study, the same formulation had been administered vaginally to 30 women, with no major safety concerns reported.

The manufacturer performed stability testing to confirm maintenance of the stated viable bacterial counts during the designated storage period. However, route-specific disintegration or dissolution kinetics in the gastrointestinal or vaginal environment were not available, and bacterial viability after oral ingestion or vaginal insertion was not directly assessed in this retrospective study.

### Endometrial microbiome test procedure

For both the first and second tests, endometrial samples were collected during the luteal phase using the same sampling procedure and the same microbiome testing method. The second sampling was performed after completion of 30 days of supplementation without a predefined washout interval. For endometrial microbiome sampling, the ectocervix was cleaned with sterile saline, and a sheathed catheter was inserted into the uterine cavity without contact with the vaginal wall or cervix. Aspirated intrauterine fluid and endometrial tissue were collected as test specimens, stored in OMNIgene-VAGINAL tubes (DNA Genotek, Ottawa, Canada), and transported to Varinos Inc. at room temperature. DNA extraction and bacterial sequence analysis were performed according to the Varinos Inc. protocol, as previously described [22]. Briefly, specimens were pretreated with MagNA Pure Bacteria Lysis Buffer (Roche, Basel, Switzerland), Proteinase K (Roche), and Lysozyme (Merck SA, Darmstadt, Germany), and DNA was extracted using the Pathogen 1000 hp 3.2 protocol of MagNA Pure 24. The V1-V2 region of the 16S rRNA gene was amplified, indexed, purified, quantified using Qubit (Thermo Fisher Scientific, Waltham, MA, USA), pooled equimolarly, and sequenced by 251-bp paired-end sequencing on MiSeq (Illumina, San Diego, CA, USA). ZymoBIOMICS Microbial Community Standard (Zymo Research, Irvine, CA, USA) and UltraPure DNase/RNase-Free Distilled Water (Thermo Fisher Scientific) were used as positive and negative controls, respectively. Sequence processing included adapter trimming with Trimmomatic, paired-read joining with EA-Utils fastq-join, quality filtering with prinseq-lite, OTU picking using QIIME 1.9.1, and taxonomic assignment by BLAST against databases based on SILVA138 and STIRRUPS; potential chimeric sequences and reagent- or environment-derived bacterial species were excluded.

### Data assessment and analysis

The patients were categorized into two groups according to the route of administration: oral and vaginal supplementation. The administration route was not randomized; it was determined as part of clinical care through patient–physician discussion/preference.

1. Baseline comparison:

Clinical background characteristics were compared between the two groups.

2. Within-group pre–post comparison and between-group comparison of improvement magnitude:

The *Lactobacillus* proportion in the endometrial microbiome was compared between the first and second tests for each group. As a secondary exploratory and descriptive outcome, we assessed the proportion of participants whose post-treatment endometrial *Lactobacillus* proportion was ≥ 80%. This threshold was selected to represent marked improvement after one month of treatment, because thresholds of approximately 80% or 90% are often used clinically to describe *Lactobacillus*-dominant or substantially improved endometrial microbiota profiles [10,14,24,26]; however, it was not used as the primary endpoint.

The increase in endometrial *Lactobacillus* proportion (post-treatment minus pre-treatment) was compared between the two groups. Because CE may be associated with endometrial dysbiosis and could influence microbiota profiles, these analyses were repeated as sensitivity analyses after excluding patients with CE (CD138-positive).

3. Microbiota composition changes:

Changes in genus- and species-level endometrial microbiota composition before and after supplementation were assessed within each group, and individual-level compositional profiles were summarized using heatmaps for visual comparison between the groups.

4. Pregnancy outcomes:

As exploratory analyses, pregnancy outcomes were summarized descriptively according to post-treatment *Lactobacillus* categories (<10%, 10% to <80%, and ≥80%). These analyses were exploratory and were not used to draw conclusions about reproductive benefit.

### Statistical analysis

All statistical analyses and figure generation were performed using R version 4.3.3. Categorical variables were compared using Fisher’s exact test. Continuous variables, including clinical characteristics and changes in the endometrial *Lactobacillus* proportion, are presented as medians (interquartile range [IQR]) and were compared between groups using the Mann–Whitney U test. Within-group changes in the proportion of *Lactobacillus* in the endometrial microbiota before and after supplementation were evaluated using the Wilcoxon signed-rank test. No formal sample size calculation was performed because this was a retrospective study using all eligible patients who underwent paired endometrial microbiome testing during the study period. Statistical significance was set at P < 0.05. All tests were two-sided.

## Results

### Patient characteristics

Between January 2022 and January 2023, 40 patients underwent an endometrial microbiome test at the initiation of infertility treatment and were identified as having a *Lactobacillus* proportion < 10%. This <10% threshold was selected pragmatically to identify patients with very low, nearly absent *Lactobacillus* abundance, rather than as a universally established diagnostic cut-off. A patient flowchart is shown in Fig 1. A second endometrial microbiome test after *Lactobacillus* supplementation was performed in 32 patients: 14 in the vaginal supplementation group and 18 in the oral supplementation group. Eight patients were excluded because they did not undergo a second endometrial microbiome test: two were lost to follow-up, and six achieved clinical pregnancy before the second test. Because endometrial microbiome testing cannot be performed during pregnancy, these patients were necessarily excluded from the paired pre–post microbiome analysis. The baseline characteristics of the two groups are summarized in Table 1, and the corresponding de-identified individual-level data are provided in S1 Dataset. No supplement-related adverse events were documented in the available medical records.

**Fig 1:**
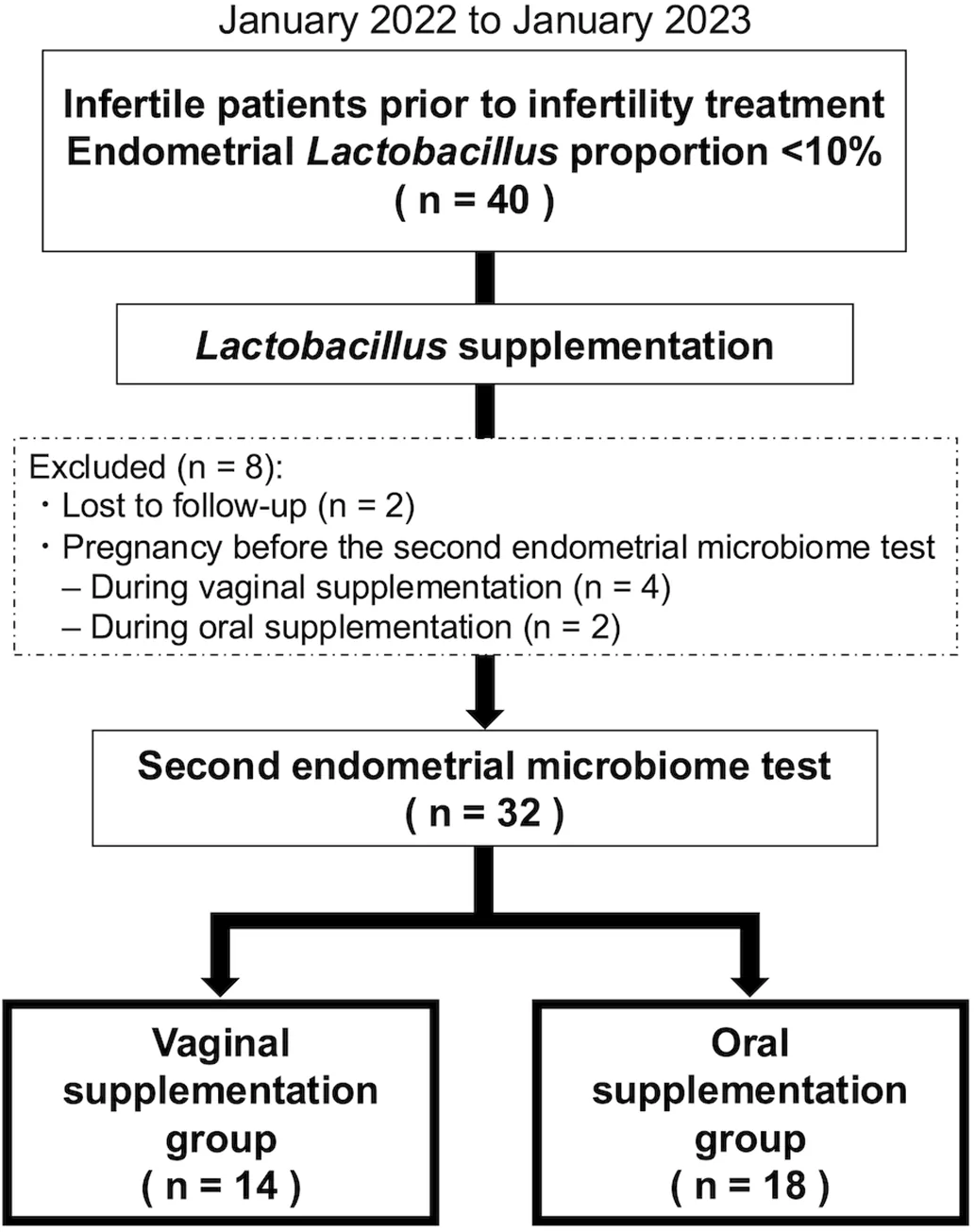
The flowchart of this study.

**Table 1:** Clinical characteristics of patients undergoing vaginal or oral *Lactobacillus* supplementation.

Characteristics	Oral group (n = 18)	Vaginal group (n = 14)	P-value
Age	33.5 [31.0–36.0]	35.0 [33.25–37.75]	0.176
Gravidity	1.0 [0.0–1.75]	1.0 [0.25–1.75]	0.810
Parity	0.0 [0.0–1.0]	1.0 [0.0–1.0]	0.388
BMI (kg/m²)	20.7 [19.4–22.2]	21.9 [19.125–23.35]	0.403
Prior infertility treatment at another clinic	38.9% (n = 7/18)	28.6% (n = 4/14)	0.712
Endometrial CD138 positivity	11.1% (n = 2/18)	35.7% (n = 5/14)	0.195

### Comparison of changes in *Lactobacillus* proportion according to the route of administration

After *Lactobacillus* supplementation, the proportion of *Lactobacillus* in the endometrial microbiota increased in both groups. In the oral supplementation group, the endometrial *Lactobacillus* proportion increased from 0.00% [0.00–0.18] to 0.30% [0.03–66.33] (p = 0.005), with a median change of +0.25 [0.03–66.33] (Fig 2(a)). In the vaginal supplementation group, the endometrial *Lactobacillus* proportion increased from 0.05% [0.00–0.20] at baseline to 99.15% [70.45–100.00] after supplementation (p = 0.001), with a median change of +98.90 [70.45–99.80] (Fig 2(b)). When the magnitude of increase was compared between the groups, the vaginal supplementation group had a significantly greater increase than the oral supplementation group (P < 0.001) (Fig 3). Furthermore, nine of 14 participants (64%) in the vaginal group had a post-treatment *Lactobacillus* proportion ≥80% compared with three of 18 participants (17%) in the oral group (P = 0.010).

**Fig 2:**
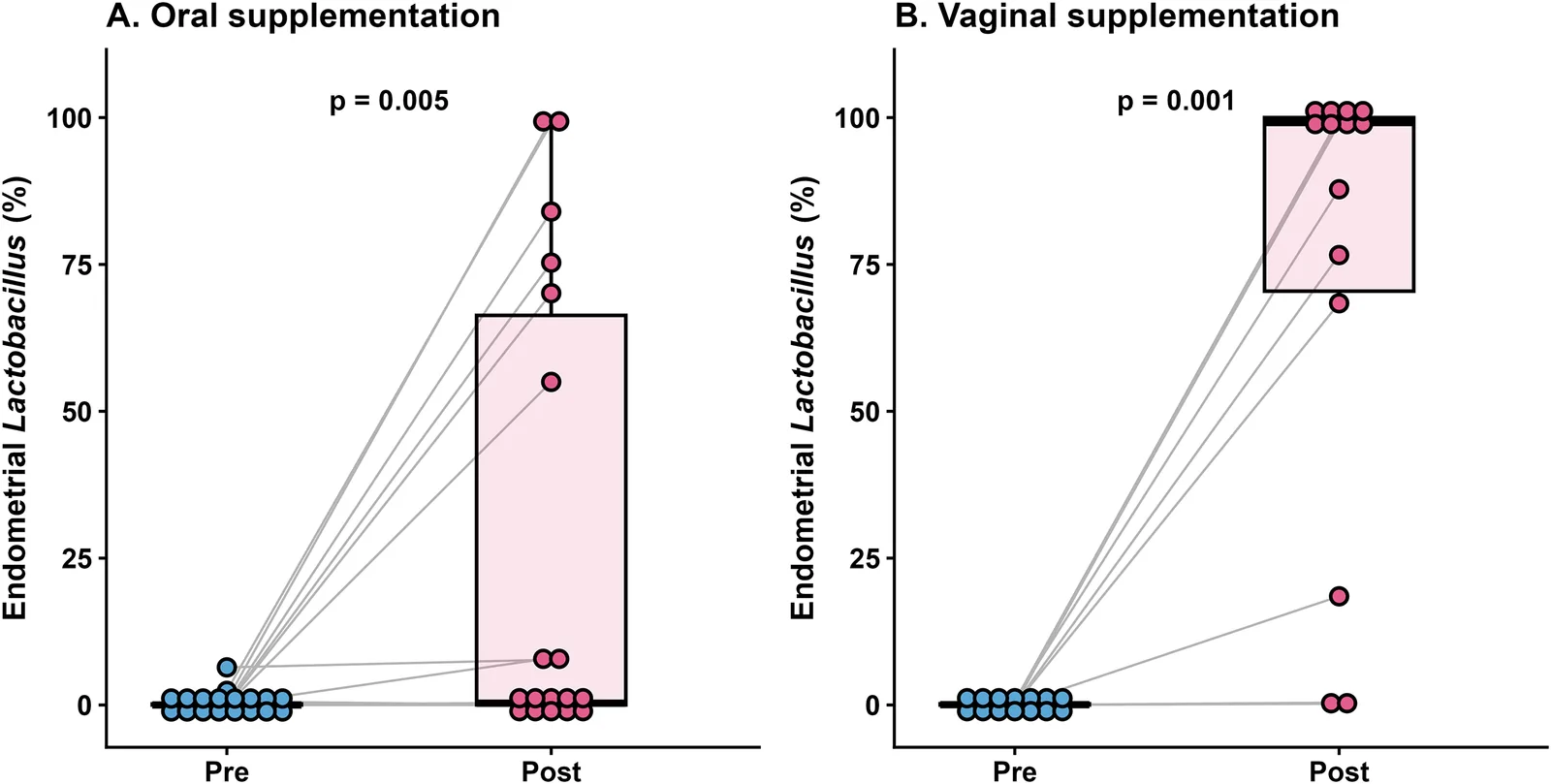
Pre- and post-treatment changes in endometrial *Lactobacillus* proportion following *Lactobacillus* supplementation. (a) oral supplementation group. (b) vaginal supplementation group. Boxplots show the median and interquartile range; points represent individual patients, and grey lines connect paired pre- and post-treatment samples. P-values were calculated using the Wilcoxon signed-rank test.

**Fig 3:**
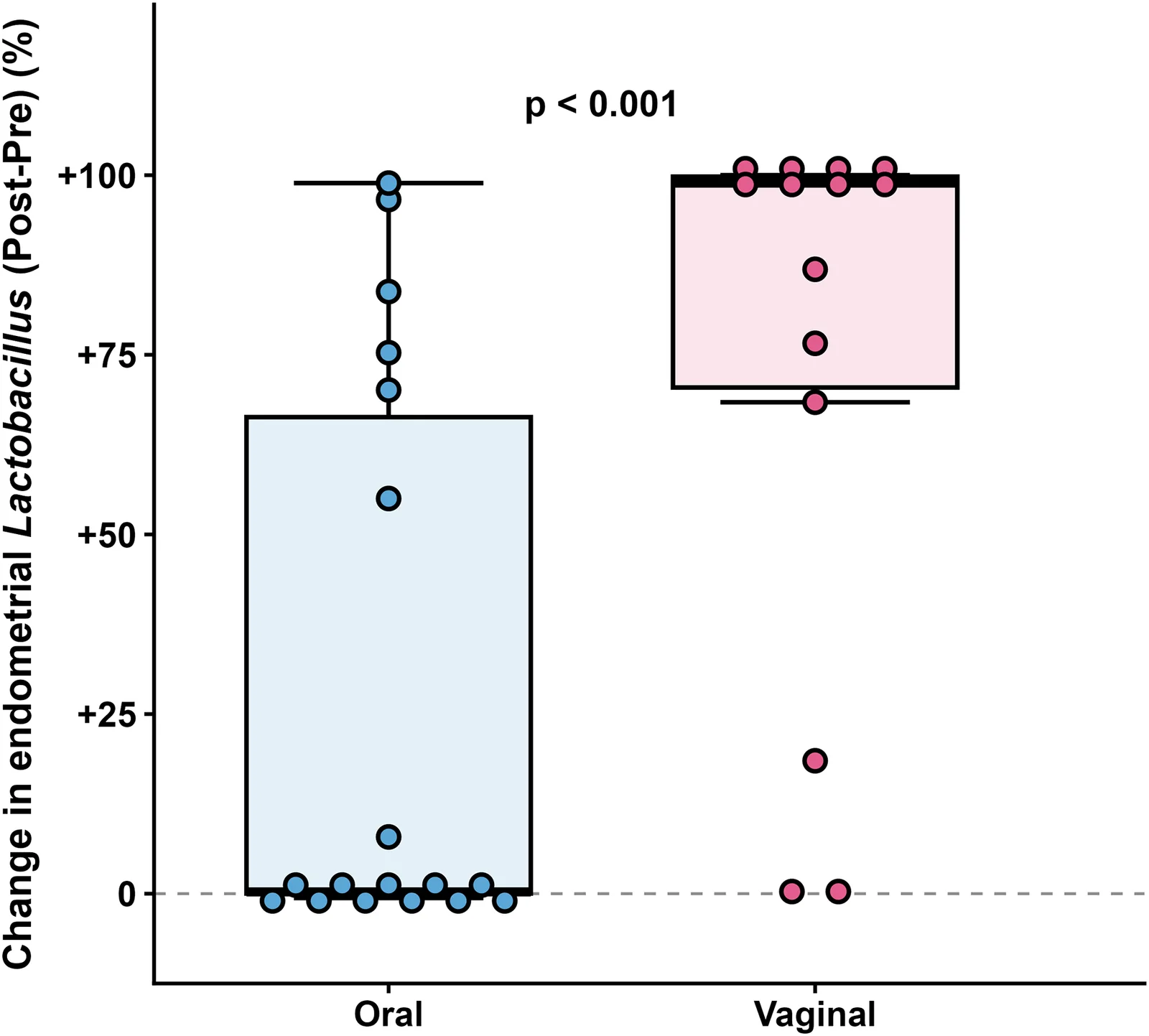
Comparison of the change in endometrial *Lactobacillus* proportion (post-treatment minus pre-treatment) between oral and vaginal supplementation groups. Boxplots show the median and interquartile range; points represent individual patients. The P-value was calculated using the Mann-Whitney U test.

A sensitivity analysis was further performed after excluding patients with CE, because CE may be associated with endometrial dysbiosis and could influence microbiota profiles. The increase in endometrial *Lactobacillus* proportion remained greater in the vaginal supplementation group (n = 9; + 86.9 [18.5–99.8]) than in the oral supplementation group (n = 16; + 0.85 [0.075–71.4]) (P = 0.020). In addition, five of nine participants (56%) in the vaginal group had a post-treatment *Lactobacillus* proportion ≥80% compared to three of 16 participants (19%) in the oral group (P = 0.087).

### Changes in the endometrial microbiota composition before and after supplementation

The individual species-level composition of the endometrial microbiota before and after supplementation is shown in the heatmap in Fig 4. In the vaginal supplementation group, post-treatment samples frequently showed high relative abundances of *Lactobacillus acidophilus* and *Lacticaseibacillus rhamnosus*, corresponding to the species included in the administered formulation. In contrast, post-treatment samples in the oral supplementation group showed more heterogeneous *Lactobacillus* species profiles, including *Lactobacillus crispatus* and *Lactobacillus iners* in some patients, which were not components of the administered formulation.

**Fig 4:**
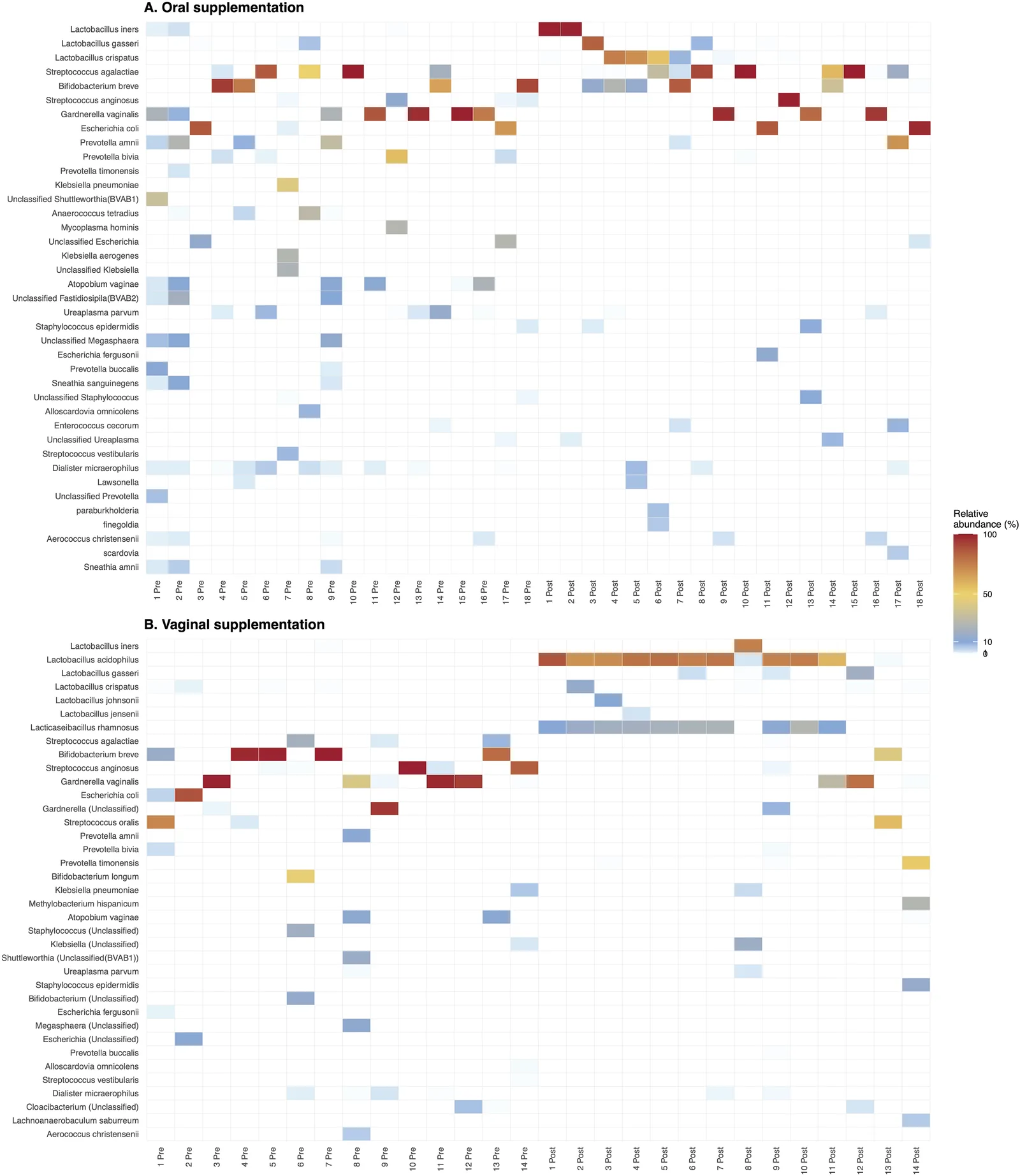
Species-level heatmap of endometrial microbiota composition before and after supplementation. (a) oral supplementation group. (b) vaginal supplementation group. Columns represent pre-treatment samples followed by post-treatment samples. Rows show species detected at ≥5% relative abundance in at least one sample or *Lactobacillus* species of clinical interest. Relative abundance (%) is shown.

### Comparison of pregnancy outcomes

Among the 32 patients who underwent two endometrial microbiome tests, 30 aged <43 years subsequently received infertility treatment (n = 30). Clinical pregnancy occurred in 10/10 patients with post-treatment *Lactobacillus* ≥80%, 6/6 patients with post-treatment *Lactobacillus* 10% to <80%, and 13/14 patients with post-treatment *Lactobacillus* <10%. Live birth occurred in 10/10, 6/6, and 8/14 patients in these respective categories.

## Discussion

This retrospective study compared route-associated changes in endometrial *Lactobacillus* abundance after oral versus vaginal *Lactobacillus* supplementation using the same *Lactobacillus*-containing probiotic formulation in women with endometrial dysbiosis (baseline endometrial *Lactobacillus* proportion <10%). Within a combined treatment regimen including standardized doxycycline/metronidazole therapy and continued oral lactoferrin, vaginal *Lactobacillus* supplementation was associated with a greater increase in endometrial *Lactobacillus* abundance than oral administration of the same formulation.

The present findings extend prior reports by comparing administration routes using the same formulation, dose, treatment duration, antibiotic regimen, and continued oral lactoferrin. This design reduces heterogeneity between route groups and allows a more focused assessment of route-associated differences, although the independent effects of antibiotics, lactoferrin, and probiotic supplementation still cannot be separated.

We defined route-associated efficiency as the magnitude of increase in endometrial *Lactobacillus* proportion from pre- to post-treatment, rather than as the achievement of a categorical threshold. The ≥ 80% threshold was used only as a secondary exploratory indicator of marked improvement because the optimal threshold for endometrial microbiome normalization remains undetermined and varies among studies [10,14,24,26]. In this framework, vaginal administration was associated with a greater increase in endometrial *Lactobacillus* abundance than oral administration within the combined treatment regimen.

The route-associated difference may be biologically plausible because vaginal supplementation delivers *Lactobacillus* directly to the lower genital tract, whereas oral supplementation likely relies on indirect gut–genital tract pathways [27–34]. Consistent with this possibility, species-level analysis showed that vaginal post-treatment samples frequently contained *Lactobacillus acidophilus* and *Lacticaseibacillus rhamnosus*, the two species included in the administered formulation. Although species-level detection alone does not prove stable colonization by viable administered organisms because strain-level confirmation and post-administration viability assessment were not performed, the possibility that vaginal administration may provide a greater treatment-associated effect than oral administration is noteworthy.

However, pregnancy outcomes were evaluated descriptively and should be interpreted with particular caution. The primary strength of this study lies in the paired observation of how the endometrial microbiome changed after supplementation using the same formulation and standardized concomitant regimen, rather than in establishing a clinically reliable reproductive threshold or proving reproductive benefit. The reproductive outcome findings should therefore be regarded as exploratory and hypothesis-generating.

This study has several limitations. First, the sample size was modest, the administration route was not randomized, and unmeasured confounding factors related to route selection or completion of a second test may have influenced the results. Second, because all participants received doxycycline, metronidazole, and continued oral lactoferrin, the findings should be interpreted as route-associated differences within a combined treatment regimen rather than as evidence for the isolated effect of probiotic therapy. Moreover, routine antibiotic use should be approached cautiously because of concerns regarding antimicrobial resistance and potential effects on the gut and reproductive tract microbiota. Future studies should therefore evaluate strategies that minimize antibiotic exposure and should specifically test whether *Lactobacillus* supplementation without concomitant antibiotics can improve endometrial dysbiosis. Third, the probiotics used in this study did not include *Lactobacillus crispatus*, which has been linked to stable, favorable genital tract microbiota states [31–33], limiting generalizability to other formulations. Finally, low-biomass endometrial microbiome profiling remains vulnerable to contamination from the vagina, cervix, reagents, or working environment despite use of a sheathed catheter, ectocervical cleaning, and sequencing controls [34]. Because no predefined washout interval was used, we could not distinguish stable endometrial colonization from transient detection of administered organisms or sampling-related contamination. Short-read 16S rRNA sequencing also cannot fully characterize strain-level differences, viability, or functional properties.

Future prospective studies should validate these findings in larger cohorts using randomized or otherwise standardized exposure protocols, predefined post-treatment timing and washout intervals, and species- or strain-level microbiome assessments. Such studies should also evaluate whether *Lactobacillus crispatus*-containing formulations produce different or more durable endometrial microbiota changes.

## Conclusion

Within a combined treatment regimen including standardized doxycycline/metronidazole therapy and continued oral lactoferrin, vaginal *Lactobacillus* supplementation was associated with a greater increase in endometrial *Lactobacillus* abundance than oral administration of the same formulation. The reproductive outcome analysis was exploratory and underpowered, and further prospective investigations are needed.

## Supporting information

S1 Dataset De-identified individual-level clinical data underlying the findings reported in this study. (XLSX)

S1 File STROBE statement. (DOCX)
