# Supplementary material for: Oral versus vaginal Lactobacillus supplementation for endometrial dysbiosis: A paired pre and post–endometrial microbiome study
Source: PLoS One. 2026 Oct 26;21(10):e0360619. doi: 10.1371/journal.pone.0360619 (PMC13704797; doi:10.1371/journal.pone.0360619)
Supplement: Supplementary file 1 [file pone.0360619.s002.docx]

STROBE Statement—checklist of items that should be included in reports of observational studies

|  | Item No. | Recommendation | Page  No. | Relevant text from manuscript |
| --- | --- | --- | --- | --- |
| **Title and abstract** | 1 | (*a*) Indicate the study’s design with a commonly used term in the title or the abstract | 1 | Title states: "Oral versus vaginal Lactobacillus supplementation for endometrial dysbiosis: a paired pre and post–endometrial microbiome study." |
|  |  | (*b*) Provide in the abstract an informative and balanced summary of what was done and what was found | 2 | Abstract summarizes the rationale, methods, key microbiome findings, and the exploratory nature of reproductive outcomes. |
|  | | | |  |
| Background/rationale | 2 | Explain the scientific background and rationale for the investigation being reported | 3–4 | Introduction describes infertility burden, uterine factors, endometrial microbiota dysbiosis (low Lactobacillus), and the rationale for evaluating Lactobacillus supplementation route. |
| Objectives | 3 | State specific objectives, including any prespecified hypotheses | 4 | Objective: to compare oral versus vaginal Lactobacillus supplementation in patients with endometrial dysbiosis using paired pre- and post-supplementation endometrial microbiome testing. |
|  | | | |  |
| Study design | 4 | Present key elements of study design early in the paper | 4 | Study design stated early: "This retrospective paired pre–post study..."; comparison between vaginal and oral supplementation groups receiving the same formulation for 30 days. |
| Setting | 5 | Describe the setting, locations, and relevant dates, including periods of recruitment, exposure, follow-up, and data collection | 4–5 | Setting and dates: patients treated at Tsubaki Women’s Clinic; screening period January 2022–January 2023; supplementation for 30 days; repeat testing after supplementation. |
| Participants | 6 | (*a*) *Cohort study*—Give the eligibility criteria, and the sources and methods of selection of participants. Describe methods of follow-up  *Case-control study*—Give the eligibility criteria, and the sources and methods of case ascertainment and control selection. Give the rationale for the choice of cases and controls  *Cross-sectional study*—Give the eligibility criteria, and the sources and methods of selection of participants | 4–5 | Eligibility and selection: infertile patients with baseline endometrial Lactobacillus <10% before any infertility treatment; excluded leiomyoma, endometrial polyps, adenomyosis, endometriosis; excluded those without a second test due to loss to follow-up or pregnancy. |
|  |  | (*b*) *Cohort study*—For matched studies, give matching criteria and number of exposed and unexposed  *Case-control study*—For matched studies, give matching criteria and the number of controls per case | N/A | Not a matched study (no matching criteria were used). |
| Variables | 7 | Clearly define all outcomes, exposures, predictors, potential confounders, and effect modifiers. Give diagnostic criteria, if applicable | 5–6 | Variables defined: exposure (route of Lactobacillus supplementation), primary microbiome outcome (change in endometrial Lactobacillus proportion), CE definition (CD138+), pregnancy outcomes (clinical pregnancy and live birth), and subgroup thresholds (Lactobacillus ≥80%, <10%). |
| Data sources/ measurement | 8* | For each variable of interest, give sources of data and details of methods of assessment (measurement). Describe comparability of assessment methods if there is more than one group | 5 | Data sources/measurement: endometrial microbiome sampling with sterile saline cleansing and a sheathed catheter; 16S rRNA gene sequencing; clinical variables obtained from medical records; CD138 immunostaining for CE. |
| Bias | 9 | Describe any efforts to address potential sources of bias | 4–5 | Bias mitigation: excluded structural pathology "to reduce potential confounding"; sampling performed with a sheathed catheter inserted without contacting vaginal wall/cervix to minimize contamination. |
| Study size | 10 | Explain how the study size was arrived at | 4–7  Fig 1 | Study size: all eligible patients within the specified period were screened (n=40); primary analysis included those with paired pre–post tests (n=32). No a priori sample size calculation was reported. |

Continued on next page

| Quantitative variables | 11 | Explain how quantitative variables were handled in the analyses. If applicable, describe which groupings were chosen and why | 5, 6 | Quantitative variables: continuous variables and Lactobacillus changes summarized as median [IQR]; change defined as post-treatment minus pre-treatment; categorical thresholds used for descriptive responders (≥80%) and pregnancy subgrouping (≥80% vs <10%). |
| --- | --- | --- | --- | --- |
| Statistical methods | 12 | (*a*) Describe all statistical methods, including those used to control for confounding | 6 | Statistical methods: Fisher’s exact test for categorical variables; Mann–Whitney U test for between-group comparisons of continuous variables; Wilcoxon signed-rank test for within-group pre–post comparisons; two-sided P<0.05; analyses performed in R 4.3.3. |
|  |  | (*b*) Describe any methods used to examine subgroups and interactions | 5, 6 | Subgroups/interactions: sensitivity analysis excluding CE (CD138-positive) patients treated with antibiotics; pregnancy outcomes compared only in patients meeting post-treatment Lactobacillus ≥80% or <10%. |
|  |  | (*c*) Explain how missing data were addressed | 5–7 | Missing data: participants without a second endometrial microbiome test were excluded from the primary analysis (reasons: loss to follow-up or pregnancy). No other missing-data handling was described. |
|  |  | (*d*) *Cohort study*—If applicable, explain how loss to follow-up was addressed  *Case-control study*—If applicable, explain how matching of cases and controls was addressed  *Cross-sectional study*—If applicable, describe analytical methods taking account of sampling strategy | 5–7 | Loss to follow-up: "Patients who did not undergo a second endometrial microbiome test... owing to loss to follow-up or pregnancy were excluded"; flow diagram (Fig 1) summarizes exclusions. |
|  |  | (*e*) Describe any sensitivity analyses | 6 | Sensitivity analyses: primary comparisons repeated after excluding CE patients who received antibiotic therapy. |
|  | | | | |
| Participants | 13* | (a) Report numbers of individuals at each stage of study—eg numbers potentially eligible, examined for eligibility, confirmed eligible, included in the study, completing follow-up, and analysed | 6–7 | Participant flow: 40 screened; 32 had repeat testing and were analyzed (vaginal n=14; oral n=18). Pregnancy and loss to follow-up prior to second testing are reported and displayed in Fig 1. |
|  |  | (b) Give reasons for non-participation at each stage | 5–7 | Reasons for non-participation: loss to follow-up and pregnancy before the second endometrial microbiome test (reported in Methods and Fig 1). |
|  |  | (c) Consider use of a flow diagram | 7 | Flow diagram provided (Fig 1: study flowchart). |
| Descriptive data | 14* | (a) Give characteristics of study participants (eg demographic, clinical, social) and information on exposures and potential confounders | 7 | Descriptive data: baseline clinical characteristics by group are presented in Table 1. |
|  |  | (b) Indicate number of participants with missing data for each variable of interest | 6 | No missing data for baseline variables. |
|  |  | (c) *Cohort study*—Summarise follow-up time (eg, average and total amount) | 5 | Pregnancy outcomes were ascertained by medical record review for up to 2 years after the second endometrial microbiome test.. |
| Outcome data | 15* | *Cohort study*—Report numbers of outcome events or summary measures over time | 6–9 | Outcome data: pre–post endometrial Lactobacillus proportions, responder proportions (>80%), species-level composition changes (Fig 4), and pregnancy outcomes including clinical pregnancy and live birth (Table 2). |
|  |  | *Case-control study—*Report numbers in each exposure category, or summary measures of exposure | N/A | Not a case-control study. |
|  |  | *Cross-sectional study—*Report numbers of outcome events or summary measures | N/A | Not a cross-sectional study. |
| Main results | 16 | (*a*) Give unadjusted estimates and, if applicable, confounder-adjusted estimates and their precision (eg, 95% confidence interval). Make clear which confounders were adjusted for and why they were included | 7–9 | Main results reported as medians [IQR] with P-values (within-group and between-group comparisons). No adjusted estimates are presented. |
|  |  | (*b*) Report category boundaries when continuous variables were categorized | 4–6, 9 | Category boundaries: baseline Lactobacillus <10% (eligibility); responder thresholds >80%; pregnancy subgrouping ≥80% vs <10%; age <43 years for pregnancy analyses. |
|  |  | (*c*) If relevant, consider translating estimates of relative risk into absolute risk for a meaningful time period | N/A | No relative risk estimates were reported; results are presented as group comparisons and proportions. |

Continued on next page

| Other analyses | 17 | Report other analyses done—eg analyses of subgroups and interactions, and sensitivity analyses | 8–9 | Other analyses: subgroup (sensitivity) analysis excluding CE patients treated with antibiotics; species-level composition changes pre–post by route (Fig 4); exploratory pregnancy subgroup analysis (Table 2). |
| --- | --- | --- | --- | --- |
|  | | | | |
| Key results | 18 | Summarise key results with reference to study objectives | 10–12 | Discussion summarizes key results aligned to objectives: greater Lactobacillus increase with vaginal vs oral supplementation using the same formulation; findings persisted after excluding CE cases; exploratory reproductive outcome findings interpreted cautiously. |
| Limitations | 19 | Discuss limitations of the study, taking into account sources of potential bias or imprecision. Discuss both direction and magnitude of any potential bias | 11–12 | Limitations include modest sample size, retrospective design, potential contamination and lack of washout interval, and limitations of short-read 16S rRNA sequencing for strain-level identification, viability, and functional characterization. |
| Interpretation | 20 | Give a cautious overall interpretation of results considering objectives, limitations, multiplicity of analyses, results from similar studies, and other relevant evidence | 10–12 | Interpretation discusses plausible mechanisms for route-dependent effects and contextualizes findings against prior literature; emphasizes hypothesis-generating nature of pregnancy outcomes. |
| Generalisability | 21 | Discuss the generalisability (external validity) of the study results | 11 | Generalisability: notes limited generalizability to other probiotic formulations (e.g., lack of L. crispatus) and need for larger prospective studies with standardized protocols. |
| Other information | |  | | |
| Funding | 22 | Give the source of funding and the role of the funders for the present study and, if applicable, for the original study on which the present article is based | N/A | The funding information will be provided in the journal’s submission system. |

*Give information separately for cases and controls in case-control studies and, if applicable, for exposed and unexposed groups in cohort and cross-sectional studies.

**Note:** An Explanation and Elaboration article discusses each checklist item and gives methodological background and published examples of transparent reporting. The STROBE checklist is best used in conjunction with this article (freely available on the Web sites of PLoS Medicine at http://www.plosmedicine.org/, Annals of Internal Medicine at http://www.annals.org/, and Epidemiology at http://www.epidem.com/). Information on the STROBE Initiative is available at www.strobe-statement.org.
