# Supplementary material for: Oral versus vaginal Lactobacillus supplementation for endometrial dysbiosis: A paired pre and post–endometrial microbiome study
Source: PLoS One. 2026 Oct 26;21(10):e0360619. doi: 10.1371/journal.pone.0360619 (PMC13704797; doi:10.1371/journal.pone.0360619)
Supplement: Supplementary file 2 [file pone.0360619.s003.docx]

June 20, 2026

Dr. António Machado
Academic Editor

PLOS One

Ref: Manuscript ID PONE-D-26-07839

Dear Dr. António Machado:

My co-authors join me in expressing our sincere appreciation to you and the reviewer for your efficient manuscript handling and helpful comments. We have critically reviewed each comment and our responses are elaborated below. The editor’s and reviewer’s comments are followed by our responses highlighted in **blue**.

***Reviewer #1:***

*Summary
This manuscript presents a retrospective paired pre–post study comparing oral versus vaginal Lactobacillus supplementation in women with endometrial dysbiosis. The study addresses a clinically relevant question and shows a greater increase in Lactobacillus with vaginal administration. However, important methodological limitations affect the strength of the conclusions.*

Thank you for this thoughtful summary and for recognizing the clinical relevance of our study. We agree that the methodological limitations affect the strength of the conclusions. We have therefore clarified the study design, strengthened the analyses and methodological descriptions, and substantially tempered the interpretation of the findings. Your constructive comments have helped us considerably improve the clarity, rigor, and overall quality of the manuscript.

*Major Comments
1. Non-randomized design introduces potential selection bias and confounding.*

Response: We appreciate the constructive comment from this reviewer. We clarified in the Methods that the administration route was not randomized and was determined as part of clinical care through patient–physician discussion/preference. We also clarified that all participants received the same standardized doxycycline/metronidazole regimen and continued oral lactoferrin, which reduces confounding by differential concomitant therapy between route groups but does not allow us to isolate the independent effect of probiotic therapy. The revised text is shown in blue in the manuscript. We have also acknowledged this point as a limitation in the revised Discussion. (**Page 5, Lines 106-109; Page 6, Lines 157-158; Page 11, Lines 296-298**)

*2. Small sample size (n=32) limits statistical power and robustness.*

Response: We appreciate the constructive comment from this reviewer. We agree that the small sample size limits statistical power and robustness. We revised the manuscript to emphasize that this was a small, retrospective, exploratory study, avoided adjusted models or confidence-interval-based effect estimation that would be unstable, and interpreted subgroup and reproductive outcome analyses as hypothesis-generating. (**Page 7, Lines 186-187; Page 11, Lines 296-298**)

*3. Risk of contamination in low-biomass endometrial microbiome analysis.*

Response: We appreciate the constructive comment from this reviewer. We agree and expanded the Limitations to address the risk of contamination in low-biomass endometrial microbiome analysis. We now state that, despite use of a sheathed catheter, ectocervical cleaning, and positive and negative controls in the sequencing workflow, contamination from the vagina, cervix, reagents, or working environment cannot be completely excluded. We also emphasized that, particularly after vaginal administration and without a predefined washout interval, stable endometrial colonization cannot be distinguished from transient detection or sampling-related contamination. The revised text is shown in blue in the Discussion. (**Page 12, Lines 307-312**)

*4. Lack of species-level microbiome resolution (e.g., L. crispatus vs L. iners).*

Response: We appreciate the constructive comment from this reviewer. We added a species-level heatmap showing individual pre- and post-treatment microbiota profiles. This analysis showed that post-treatment samples in the vaginal group frequently contained *Lactobacillus acidophilus* and *Lacticaseibacillus rhamnosus*, corresponding to the species included in the administered formulation, whereas oral post-treatment samples showed more heterogeneous *Lactobacillus* species profiles, including *Lactobacillus crispatus* and *Lactobacillus iners* in some patients. We revised the Discussion to state that species-level detection alone does not prove stable colonization by viable administered organisms because strain-level confirmation and post-administration viability assessment were not performed, while also noting that the possibility of a greater treatment-associated effect with vaginal administration is noteworthy. The revised text and Fig 4 are shown in blue/revised form in the manuscript. (**Fig. 4; Pages 9-10, Lines 240-246; Page 11, Lines 283-289**)

*5. Overinterpretation of exploratory clinical outcomes (live birth rates).*

Response: We appreciate the constructive comment from this reviewer. We agree and substantially revised the reproductive outcome section. We removed Table 2, summarized reproductive outcomes descriptively in the text, included the intermediate post-treatment *Lactobacillus* category. We also emphasized that these analyses were exploratory and underpowered. In the Discussion, we further clarified that the primary strength of the study lies in the paired observation of endometrial microbiome changes after supplementation, rather than in establishing a clinically reliable reproductive threshold or proving reproductive benefit. (**Page 7, Lines 177-179; Page 10, Lines 256-259; Page 11, Lines 290-295**)

*6. Arbitrary threshold of ≥80% Lactobacillus.*

Response: We appreciate the constructive comment from this reviewer. We clarified that the primary endpoint was the continuous pre-to-post change in endometrial *Lactobacillus* proportion, not achievement of a categorical threshold. The ≥80% threshold was used only as a secondary exploratory and descriptive indicator of marked improvement after one month of treatment, because thresholds around 80% or 90% are commonly used clinically to describe *Lactobacillus*-dominant or substantially improved endometrial microbiota profiles. The revised text is shown in blue in the Methods and Discussion. (**Page 7, Lines 164-167; Page 11, Lines 274-278**)

*7. Use of mean ± SD despite non-normal distributions.*

Response: We appreciate the constructive comment from this reviewer. We removed mean ± SD values from the main Abstract and Results and now present continuous microbiome outcomes consistently as medians with interquartile ranges, matching the nonparametric statistical analyses. The revised text is shown in blue in the manuscript. (**Pages 2, Lines 33-35; Page 9, Lines 213-216**)

*Minor Comments
1. Clarify timing of sampling and menstrual cycle phase.*

Response: We appreciate the constructive comment from this reviewer. We clarified that both the first and second endometrial microbiome samples were collected during the luteal phase. We also stated that the same sampling procedure and microbiome testing method were used for both tests. The revised text is shown in blue in the Methods. (**Page 6, Lines 136-137**)

*2. Address antibiotic treatment as potential confounder.*

Response: We appreciate this important comment and apologize for the inaccurate description in the original manuscript, which incorrectly implied that only some participants received antibiotic treatment. After rechecking the treatment protocol and clinical records, we confirmed that all participants received the same standardized doxycycline/metronidazole regimen and continued oral lactoferrin, irrespective of the route of probiotic administration. We have corrected this information throughout the revised manuscript. We revised the Methods, Results, Discussion, Limitations, Abstract, and Conclusion to make clear that the study evaluates the route-associated additional contribution of vaginal versus oral Lactobacillus supplementation within this combined treatment regimen. The revised text is shown in blue in the manuscript. (**Page 2, Lines 27-28, 39; Page 5, Lines 106-109; Page 10-11, Lines 265-273, Pages 11-12, Lines 298-305; Page 12, Lines 318-319**)

*3. Data availability statement does not comply with PLOS requirements.*

Response: We appreciate the constructive comment from this reviewer. To improve transparency and comply with the PLOS Data Policy, we have prepared a de-identified individual-level dataset containing the clinical data underlying the findings reported in the manuscript. This dataset has been uploaded as S1 Dataset. We have revised the Data Availability Statement accordingly. (**Page 12, Lines 324-325**)

*4. Improve figure clarity and statistical annotation.*

Response: We appreciate the constructive comment from this reviewer. We revised Figs 2 and 3 to improve clarity, readability, and statistical annotation. Fig 2 was redrawn as paired boxplots with individual patient points and grey connecting lines, with Wilcoxon signed-rank test P-values shown. Fig 3 was redrawn as a boxplot of pre-to-post change with individual patient points, with the Mann-Whitney U test P-value shown. We also reformatted the figures using clearer dot overlays, median/IQR boxplots, simplified P-value labels, bold Arial axis text, and consistent color coding. The revised figure legends specify that boxplots show medians and interquartile ranges (**Fig. 2; Fig. 3; Page 9, Lines 228-237**)

*5. Minor language refinements needed.*

Response: We appreciate the constructive comment from this reviewer. We carefully reviewed the manuscript and corrected minor typographical and grammatical errors.

*Strengths
- Paired pre–post design
- Same probiotic formulation in both groups
- Clinically relevant topic
- Generally clear writing and transparent discussion
Recommendation
Major Revision
Suggested Closing Statement
This study addresses an important and clinically relevant question using a paired design and standardized intervention. However, significant limitations related to its retrospective, non-randomized design, small sample size, and methodological challenges inherent to low-biomass microbiome analysis limit the strength of the conclusions. I recommend major revision.*

***Reviewer #2:***

*The authors proposed a retrospective study that compares the effects of oral and vaginal Lactobacillus supplementation in patients with endometrial microbiota dysbiosis who underwent endometrial microbiome testing before and after supplementation. The idea is very attractive but not completely original. Several reports already studied both administration strategies. Authors must improve the manuscript by rewriting it, checking spelling mistakes, and providing more data. In general, the study is interesting but lacks sufficient data to support the main findings.*
Thank you for your careful and critical assessment of our study. We appreciate your recognition of the clinical interest of this topic and agree that the original manuscript required additional clarification and supporting information. In response, we extensively revised the manuscript, added methodological and individual-level data, corrected language and typographical errors, and moderated the interpretation of the findings. Your comments have helped us substantially improve the rigor, transparency, and overall quality of the manuscript.

*Comments to authors:
1. Authors need to use the new taxonomic nomenclature for lactic acid bacteria.*

Response: We appreciate the constructive comment from this reviewer. As your indication, we updated the taxonomic nomenclature for the administered probiotic strain from *Lactobacillus rhamnosus* HN001™ to *Lacticaseibacillus rhamnosus* HN001™. (**Page 5, Line 121; Page 10, Line 242; Page 11, Line 285**)

*2. Line 100: What material is the administered capsule made of? How do you know the capsule material is suitable for oral and vaginal administration? Was bacterial viability assayed? The results of the capsule's resistance to the digestive tract or vaginal environment should be included. A more detailed description of the capsule (manufacturing process, composition, etc.) is necessary. Please include the corresponding information.*

Response: We appreciate the constructive comment from this reviewer. We obtained additional product information and added the available details to the Methods. The same capsule formulation was used for oral and vaginal administration. The capsule shell consisted mainly of hydroxypropyl methylcellulose, with small amounts of carrageenan and potassium chloride and trace dimethylpolysiloxane and sorbitan fatty acid ester; excipients included starch, calcium stearate, and silicon dioxide, and no special coating was used. Oral use was supported by Japanese Pharmacopoeia disintegration testing, whereas vaginal use was supported by prior use as a vaginal preparation in 30 women without major reported problems. Manufacturer stability testing was performed to support viable bacterial counts during storage. However, specific data on capsule resistance, disintegration, or dissolution in the digestive or vaginal environment, and bacterial viability after oral or vaginal administration, were not available in this retrospective dataset. We added this information and limitation to the Methods. (**Pages 5-6, Lines 120-134**)

*3. Line 107: Why were patients receiving antibiotics not excluded?*

Response: We appreciate this important comment and apologize for the inaccurate description in the original manuscript, which incorrectly implied that only some participants received antibiotic treatment. After rechecking the treatment protocol and clinical records, we confirmed that all participants received the same standardized doxycycline/metronidazole regimen and continued oral lactoferrin, irrespective of the route of probiotic administration for low endometrial Lactobacillus abundance. We have corrected this information throughout the revised manuscript. Therefore, there was no antibiotic-free subgroup to exclude. We revised the Methods and Discussion accordingly and reframed the study as a comparison of oral versus vaginal Lactobacillus supplementation added to the same concomitant regimen. (**Page 2, Lines 27-28, 39; Page 5, Lines 106-109; Page 10 and 11, Lines 265-273, Pages 11-12, Lines 298-305; Page 12, Lines 318-319**)

*4. Line 112: It is not indicated when the samples were withdrawn. Was the time for sample collection the same for all patients?*

Response: We appreciate the constructive comment from this reviewer. We clarified the sampling timing. Both first and second samples were collected during the luteal phase, and the second sample was collected after completion of 30 days of supplementation. We also noted that no predefined washout interval was included before the second sampling and added this point to the Limitations. The revised text is shown in blue in the manuscript. (**Page 6, Lines 136-138**)

*5. Line 127: Sentence is not clear. The results presented in the paper do or do not include patients who received antibiotic treatment.*

Response: We appreciate the constructive comment from this reviewer. We revised the Methods to clarify that the primary analyses included all eligible patients and that all participants received standardized doxycycline/metronidazole therapy and continued oral lactoferrin (This point is addressed in our response to Comment 3). We also clarified that the CE-exclusion analysis was a sensitivity analysis addressing the possible influence of CE on endometrial dysbiosis, not an antibiotic-exclusion analysis. The revised text is shown in blue in the manuscript. (**Page 7 169-170**)

*6. Line 134: Why did the authors choose 10% and 80% as cut-off points?*

Response: We appreciate the constructive comment from this reviewer. We clarified the rationale for both cut-offs. The baseline <10% threshold was selected pragmatically to identify patients with very low, nearly absent Lactobacillus abundance, rather than as a universally established diagnostic cut-off. The post-treatment ≥80% threshold was used only as a secondary exploratory and descriptive indicator of marked improvement after one month of treatment, because thresholds around 80% or 90% are commonly used clinically to describe *Lactobacillus*-dominant or substantially improved endometrial microbiota profiles. The revised text is shown in blue in the Methods and Discussion. (**Page 7, Lines 164-167; Page 11, Lines 274-278**)

*7. Lines 141-143: It is not clear why the authors performed a statistical study, but they showed data not used for statistics. Please use the same data for both the results and statistical study.*

Response: We appreciate the constructive comment from this reviewer. We revised the Results so that the data presented in the text correspond to the data used for statistical testing. Median values and interquartile ranges are now used consistently for both descriptive reporting and nonparametric statistical comparisons, and mean ± SD values were removed from the main text. The revised text is shown in blue in the manuscript. (**Page 2, Lines 33-35; Page 9, Lines 213-216**)

*8. Line 163: It is not indicated if assayed lactobacilli are resistant or sensitive to the antibiotics.*

Response: We appreciate this important comment. We contacted the manufacturer to obtain additional information regarding the viability of the administered probiotic strains. According to the manufacturer, stability testing was performed to confirm maintenance of the stated viable bacterial counts during the designated storage period. However, the susceptibility of the administered strains to doxycycline or metronidazole was not assessed in the present study, and corresponding susceptibility data were not available from the manufacturer. In addition, bacterial viability after oral or vaginal administration, including under concomitant antibiotic exposure, was not evaluated. We have clarified these points in the revised Methods and Discussion. Because the survival and activity of the administered strains during antibiotic treatment could not be determined, we cannot isolate the independent effect of probiotic supplementation. We therefore reframed our findings as a route-associated difference observed within a combined treatment regimen including standardized doxycycline/metronidazole therapy and continued oral lactoferrin, rather than as evidence of an isolated probiotic effect. (**Pages 5-6, Lines 131-134**)

*9. Lines 163-170: 6/14 patients had less than 90% of the Lactobacillus proportion, and in the oral group, 7/18 patients had 5-90%. Although authors indicate differences are statistically significant, data in the manuscript are different. Authors must present the results according to the statistical studies performed.*

Response: We appreciate the constructive comment from this reviewer. We carefully rechecked the individual-level data and all statistical analyses. The statistical significance reported for the primary analyses was based on continuous *Lactobacillus* proportion data rather than on the numbers of patients falling within specific percentage ranges. Specifically, within-group pre- to post-treatment changes were evaluated using the Wilcoxon signed-rank test, and the magnitude of change was compared between the oral and vaginal groups using the Mann–Whitney U test.
In contrast, the categorical analyses based on the numbers of patients with post-treatment *Lactobacillus* proportions <10% or ≥80% were conducted separately using Fisher’s exact test. Thus, the continuous and categorical outcomes represent distinct analyses and should not be interpreted interchangeably. The numbers based on the 5% and 90% boundaries mentioned by the reviewer were not used for the statistical tests reported in the manuscript.
To eliminate potential confusion caused by overlapping data points in the original figures, we have replaced the figures with box plots in which all individual data points are clearly visible. We have also revised the Results and figure legends to clearly identify the outcome and statistical test corresponding to each reported P value. (**Fig. 2; Fig. 3; Page 9, Lines 213-220, 228-237**)

*10. Line 220: The experimental data are not enough to support this statement.*

Response: We appreciate this constructive comment. We revised the relevant statement and substantially tempered the interpretation. The findings are now described as route-associated differences observed within a combined treatment regimen, without implying a definitive or isolated probiotic treatment effect. We also retained the sensitivity analysis excluding patients with chronic endometritis as supportive information only, because the resulting subgroup sizes were small and did not permit robust inference. Accordingly, this analysis is presented as exploratory and is not used as the primary basis for our conclusions. The revised text is shown in blue in the Discussion. (**Pages 10–11, Lines 262–273**)

*11. Lines 232-234: Why did the author consider antibiotic treatment not important? Furthermore, within a group, some patients received antibiotic treatment but others did not. In this case, not all patients were in the same conditions and therefore should not be compared to the rest of the patients.*

Response: We appreciate the constructive comment from this reviewer. We agree with the reviewer and this interpretation. Antibiotic treatment should not be considered unimportant; rather, all participants received the same standardized antibiotic regimen and continued oral lactoferrin. We apologize for the inaccurate description in the original manuscript, which incorrectly implied that only some participants received antibiotic treatment. We therefore removed statements suggesting that the findings reflect Lactobacillus supplementation alone and added a limitation that the independent effects of antibiotics, lactoferrin, and Lactobacillus supplementation could not be separated. We also added that routine antibiotic use should be approached cautiously because of concerns regarding antimicrobial resistance and potential effects on the gut and reproductive tract microbiota, and that future studies should evaluate strategies that minimize antibiotic exposure, including Lactobacillus supplementation without concomitant antibiotics. (**Page 2, Lines 27-28, 39; Page 5, Lines 106-109; Page 10-11, Lines 265-273, Pages 11-12, Lines 298-305; Page 12, Lines 318-319**)

*12. Line 234: This statement is not correct because authors did not indicate whether lactobacilli are sensitive or resistant to the antibiotic treatment.*

Response: We appreciate the constructive comment from this reviewer. We agree with the reviewer. The susceptibility of the administered *Lacticaseibacillus rhamnosus* HN001 and *Lactobacillus acidophilus* LA-14 strains to doxycycline and metronidazole was not assessed in this study, and corresponding susceptibility data were not available from the manufacturer. The manufacturer’s stability testing confirmed maintenance of viable bacterial counts during storage but did not evaluate viability under antibiotic exposure. We therefore removed the unsupported statement and no longer infer that the observed microbiome changes reflect an isolated probiotic effect or that the administered strains remained viable during antibiotic treatment. The findings are now interpreted only as route-associated differences observed within a combined treatment regimen including standardized antibiotic therapy, continued oral lactoferrin, and probiotic supplementation. We have also acknowledged the inability to determine antibiotic susceptibility and post-administration viability as a limitation in the revised Discussion. (**Pages 5-6, Lines 131-134; Page 11-12, Lines 298-301**)

*13. Line 237: It is not fully understood how the authors defined "efficiency," and which was the cut-off point?*

Response: We appreciate the constructive comment from this reviewer. We clarified that route-associated “efficiency” was operationally defined as the magnitude of increase in endometrial Lactobacillus proportion from pre- to post-treatment, rather than as the achievement of a categorical cut-off. The revised text is shown in blue in the Discussion. (**Page 11, Lines 274-275**)

*14. Lines 239-240: The interpretation is biased due to a lack of clarity as previously indicated.*

Response: We appreciate the constructive comment from this reviewer. We agree that the original wording could be interpreted as implying superiority and was not sufficiently cautious. We therefore removed the term “more efficient” and revised the statement to describe only the observed route-associated difference. (**Page 11, Lines 278-280**)

*15. Line 254: The results are not enough to support this statement.*

Response: We appreciate the constructive comment from this reviewer. We revised the reproductive outcome interpretation to make clear that pregnancy outcomes were evaluated descriptively and should be interpreted with particular caution. We also clarified that the primary strength of the study is the observation of endometrial microbiome changes after supplementation, whereas reproductive outcome findings remain exploratory and hypothesis-generating. (**Page 11, Lines 290-295**)

*16. Line 256: Why do authors refer to subclinical inflammation if it has not been mentioned before as a factor? No inflammatory or immunological parameters were evaluated.*

Response: We appreciate the constructive comment from this reviewer. We removed the statement referring to improved local immune homeostasis or reduced subclinical inflammation.

*17. Lines 259-261: This sentence highlights the lack of results to support the interpretations.*

Response: We appreciate the constructive comment from this reviewer. We revised this passage to avoid overinterpretation. The reproductive outcome findings are now described as exploratory and underpowered, and the Discussion clarifies that the study was primarily designed to observe route-associated endometrial microbiome changes after supplementation rather than to establish reproductive benefit. (**Page 11, Lines 290-295**)

*18. Lines 282-284: The limitations of the study are too significant to ignore them; therefore, the conclusions are not fully supported.*

Response: We appreciate the constructive comment from this reviewer. We revised the Conclusion to reflect the study limitations more clearly. The conclusion states that vaginal administration was associated with a greater increase in endometrial Lactobacillus abundance within a combined treatment regimen, while reproductive outcomes remain exploratory and underpowered. We also revised the Discussion to emphasize that the study’s main strength is the paired observation of endometrial microbiome changes after supplementation. (**Page 11, Lines 291-294; Page 12, Lines 318-322**)

***Reviewer #3:***

*The manuscript is significant for its comparison of Lactobacillus supplementation administered orally versus vaginally in patients with endometrial dysbiosis, and it further contributes by providing a before-and-after comparison of treatment outcomes. However, the study has several limitations not mentioned in the text, such as a small sample size, the absence of clear randomization, and its single-center, retrospective design. Additionally, there are potential selection biases, cases of antibiotic use, missing information (primarily in the methodology and results), limitations inherent to 16S rRNA analysis at the genus level, risk of sample contamination, etc. Therefore, major changes are suggested, and the results and conclusions should be formulated with greater caution; the methodology and results should be described in greater detail, and additional analyses should be conducted. Specifically, I suggest the following:

In the abstract, infertility is not linked to the rationale for conducting the study; I recommend improving the wording and also specifying the type of sample used to compare the microbiome*

Response: We appreciate the constructive comment from this reviewer. We revised the Abstract to more clearly link infertility, impaired endometrial receptivity, endometrial dysbiosis, and the rationale for comparing administration routes. We also specified that the microbiome comparison was based on endometrial samples, including aspirated intrauterine fluid and endometrial tissue. The revised text is shown in blue in the Abstract. (**Page 2, Lines 25, 29-30**)

*Lines 52–58: The importance of the study is established here, but there are only three references in the entire paragraph. Please provide stronger support for the claims made.*

Response: We appreciate the constructive comment from this reviewer. We added two references to support the background statements regarding endometrial microbiota, Lactobacillus-dominant and non-Lactobacillus-dominant microbiota, and the prevalence of endometrial dysbiosis among infertile women. The revised citations are shown in blue in the Introduction. (**Page 3, Lines 61-62**)

*Lines 65–66: Include references*

Response: We appreciate the constructive comment from this reviewer. We added two references to support the background statements regarding endometrial microbiota, Lactobacillus-dominant and non-Lactobacillus-dominant microbiota, and the prevalence of endometrial dysbiosis among infertile women. The revised citations are shown in blue in the Introduction. (**Page 3, Line 73**)

*Line 70: The term “dysbiosis” is specific to the microbiota; please change “endometrial microbiota dysbiosis” to “endometrial dysbiosis” and review this throughout the manuscript.*

Response: We appreciate the constructive comment from this reviewer. We changed “endometrial microbiota dysbiosis” to “endometrial dysbiosis” and reviewed the manuscript for the same wording. The revised wording is shown in blue in the Introduction. (**Page 4, Line 77**)

*Lines 69–73: The objective is written in several sentences, which makes it confusing; please rewrite it as a single paragraph with an appropriate structure to ensure clarity.*

Response: We appreciate the constructive comment from this reviewer. We rewrote the objective as a single clear paragraph: " This study aimed to compare route-associated changes in endometrial Lactobacillus abundance after oral versus vaginal Lactobacillus supplementation, administered as an adjunct to standardized doxycycline/metronidazole therapy and continued oral lactoferrin, in patients with endometrial dysbiosis who underwent paired pre- and post-supplementation endometrial microbiome testing”. (**Pages 3-4, Lines 74-78**)

*Lines 77–84: Include the date on which the ethics committee approved the project.*

Response: We appreciate the constructive comment from this reviewer. We added the ethics committee approval date. The study protocol was approved by the Research Ethics Committee of the Faculty of Medicine at the University of Tokyo on 13 November 2025. The revised text is shown in blue in the Methods. (**Page 4, Lines 89**)

*Lines 91–92: Explain who linked the information to the records (since it is later explained that it was not the authors).*

Response: We appreciate the constructive comment from this reviewer. We clarified the data-handling procedure. MN and AI extracted data from the electronic medical records at Tsubaki Women’s Clinic and anonymized the dataset before analysis; DH then performed the statistical analyses using the anonymized dataset. The revised text is shown in blue in the Methods. (**Page 4, Lines 96, 99-100**)

*Provide information on the excluded patients (due to lack of follow-up and pregnancy) because, given the small sample size, pregnancy in particular would be a relevant clinical outcome that could alter the interpretation of results regarding the treatment’s effectiveness. Include this information in the diagram (Figure 1).*

Response: We appreciate the constructive comment from this reviewer. We agree that the excluded patients should be described because of the small sample size. Figure 1 shows that eight patients were excluded: two were lost to follow-up and six achieved clinical pregnancy before the second endometrial microbiome test. We added this information to the Results and clarified that, among the six pregnancy-related exclusions, two had received oral supplementation and four had received vaginal supplementation. Because endometrial microbiome testing cannot be performed during pregnancy, these patients were necessarily excluded from the paired pre–post microbiome analysis. The revised text is shown in blue in the manuscript. (**Fig 1; Pages 8, Lines 198-203**)

*Line 94: Indicate whether the pre-treatment microbiome results were obtained using the same sample and technique as the post-treatment results.*

Response: We appreciate the constructive comment from this reviewer. We clarified that the pre- and post-treatment microbiome results were obtained using the same specimen type, sampling procedure, and 16S rRNA sequencing-based microbiome testing method. The revised text is shown in blue in the Methods. (**Page 6, Lines 136-137**)

*Lines 98–100: Explain whether the capsules contained any other excipients, whether there was a specific coating, provide further details on the composition of the capsules, and highlight whether there were any differences between those administered orally and those administered vaginally. It is also necessary to detail the route and dosage of administration for both routes, particularly what recommendations were given for administration. Another important point would be to clarify how the route of administration was chosen for each patient—was it randomized?*

Response: We appreciate the constructive comment from this reviewer. The same probiotics without any route-specific modification, was used for both oral and vaginal administration. We added additional capsule information, including the absence of route-specific capsule differences, capsule composition, lack of special coating, daily dose and route, and the nonrandomized patient–physician discussion/preference-based route selection. We have also acknowledged this point as a limitation in the revised Discussion. (**Pages 5-6, Lines 120-134; Page 11, Lines 296-298**)

*It is noted that some patients received antibiotic treatment, and this treatment may have altered the endometrial and vaginal microbiota; therefore, this should be treated as a relevant confounder. A table could be included showing individual patient information to identify who used antibiotics, which route of administration was used for the Lactobacillus supplement, pre- and post-treatment microorganism percentages, and reproductive outcomes.*

We appreciate this important comment and apologize for the inaccurate description in the original manuscript, which incorrectly implied that only some participants received antibiotic treatment. After rechecking the treatment protocol and clinical records, we confirmed that all participants received the same standardized doxycycline/metronidazole regimen and continued oral lactoferrin, irrespective of the route of probiotic administration for low endometrial Lactobacillus abundance. We have corrected this information throughout the revised manuscript. Therefore, there was no antibiotic-free subgroup to exclude. We revised the Methods and Discussion accordingly and reframed the study as a comparison of oral versus vaginal Lactobacillus supplementation added to the same concomitant regimen. (**Page 2, Lines 27-28, 39; Page 5, Lines 106-109; Page 10 and 11, Lines 265-273, Pages 11-12, Lines 298-305; Page 12, Lines 318-319**)

As indicated, we also added a species-level heatmap showing individual pre- and post-treatment microbiota profiles. (**Fig 4**)

*Lines 112–116: Detail the extraction procedure, sequencing (amplified region, primers, quality results, read depth, controls), and bioinformatic analyses performed*

Response: We appreciate the constructive comment from this reviewer. In response to the reviewer’s comment, we contacted the testing company and added all available methodological details regarding DNA extraction, sequencing, quality control, read depth, controls, and bioinformatic analyses, to the extent that this information could be disclosed by the company. (**Page 6, Lines 141-154**)

*Line 179: “Lactobacillus” should be in italics; please check that this error does not appear elsewhere in the manuscript, including tables and figures
Explain what software was used to create the figures
I recommend avoiding interpreting the lack of significance in Table 1 as due to small sample sizes.*

Response: We appreciate the constructive comment from this reviewer. We reviewed the entire manuscript, including the tables, figures, and figure captions, and italicized *Lactobacillus* wherever necessary. The term was not modified in reference titles, which were retained as originally published. The corrections are shown in blue in the revised manuscript.
We also clarified in the Statistical Analysis section that figure generation was performed using R version 4.3.3.
Regarding Table 1, we agree that the absence of statistical significance should not be interpreted as evidence of equivalence between the groups. We therefore revised the text to state only that the participant characteristics are summarized in Table 1, without interpreting the non-significant results. (**Page 7, Line 181**)

*Table 2 contains little data that could be explained in the text; I suggest removing it.*

Response: We appreciate the constructive comment from this reviewer. We removed Table 2 and summarized reproductive outcomes briefly in the Results text. (**Page 10, Lines 256-259**)

*The risk of bias acknowledged by the authors may be a significant limitation and could even directly affect the study’s main conclusion; therefore, statements should be tempered, and it should be clarified what could not be demonstrated.*

We appreciate the constructive comment from this reviewer. We revised the Conclusion to reflect the study limitations more clearly. The conclusion states that vaginal administration was associated with a greater increase in endometrial Lactobacillus abundance within a combined treatment regimen, while reproductive outcomes remain exploratory and underpowered. We also revised the Discussion to emphasize that the study’s main strength is the paired observation of endometrial microbiome changes after supplementation. We also added: imitations and future perspective. (**Pages 11-12, Lines 291-316; Page 12, Lines 318-322**)

*The statistical analyses should be more robust and should include confidence intervals; it is also important to justify the sample size. Median values or effect estimators with confidence intervals, hazard ratios, or sensitivity differences with adjusted models could be included.*

Response: We appreciate the constructive comment from this reviewer. We revised continuous variables to be reported as medians with interquartile ranges and used nonparametric tests throughout. Because this was a small, retrospective, exploratory study using all eligible paired cases, we did not perform adjusted models or confidence-interval-based effect estimation, as these analyses would be unstable and prone to overfitting. We added that no formal sample size calculation was performed because the study included all eligible patients who underwent paired endometrial microbiome testing during the study period. The revised text is shown in blue in the Statistical analysis section. (**Page 7, Lines 181-188**)

*The section on reproductive outcomes should be presented as exploratory analyses because patients with intermediate values are excluded; alternatively, the results for this group could be included to provide more comprehensive analyses indicating what occurred with patients in the intermediate group.*

Response: We appreciate the constructive comment from this reviewer. We agree. We revised the reproductive outcome section as an exploratory descriptive analysis and included the intermediate post-treatment Lactobacillus category. Clinical pregnancy occurred in 10/10, 6/6, and 13/14 patients, and live birth occurred in 10/10, 6/6, and 8/14 patients in the ≥80%, 10% to <80%, and <10% categories, respectively. We further clarified that these reproductive outcomes are secondary and that the study’s main strength is the paired assessment of endometrial microbiome changes. (**Page 10, Lines 255-259**)

*Please avoid any causal inferences throughout the manuscript; carefully review the wording to ensure this. In particular, the conclusions have a causal tone that emphasizes the superiority of vaginal administration. Although the finding is interesting, the study’s limitations do not allow for a definitive conclusion that vaginal administration is superior. Please rephrase the conclusion and similar statements throughout the manuscript.*

Response: We appreciate the constructive comment from this reviewer. We carefully reviewed the wording and revised causal or overly definitive language throughout the manuscript. In particular, we reframed the findings as route-associated differences within a combined treatment regimen, removed the causal discussion of subclinical inflammation, and weakened the Conclusion to avoid claiming definitive superiority of vaginal administration. The revised text is shown in blue in the manuscript. (**Pages 11-12, Lines 291-316; Page 12, Lines 318-322**)

*Indicate whether or not there were any adverse effects associated with the administration of the supplement; also explain whether the capsules’ viability was assessed and whether all necessary authorizations were obtained for both administration routes.*

Response: We appreciate the constructive comment from this reviewer. We added the available product information to the Methods. The manufacturer reported prior use of the capsule as a vaginal preparation in 30 women without major reported problems, and no special coating was used. Manufacturer stability testing was performed to support viable bacterial counts during storage; however, bacterial viability after oral or vaginal administration was not assessed in this retrospective study. Systematic adverse-event collection was not part of this retrospective dataset, and detailed post-administration viability testing was not available. We therefore avoided overinterpreting stable colonization or viable organism persistence and explicitly noted the limits of viability assessment.
No supplement-related adverse events were documented in the available medical records, and this information has been added to the Results.
We also clarified that the study was approved by the institutional ethics committee.
(**Pages 5-6, Lines 120-134; Page 8, Line 203; Page 11, Lines 281-289**)

*There is a discrepancy regarding data availability, as the authors state that all data are available without restriction; however, some information cannot be shared publicly due to the nature of the study.*

Response: We appreciate the constructive comment from this reviewer. To improve transparency and comply with the PLOS Data Policy, we have prepared a de-identified individual-level dataset containing the clinical data underlying the findings reported in the manuscript. This dataset has been uploaded as S1 Dataset. We have revised the Data Availability Statement accordingly. (**Page 12, Lines 324-325**)

*It is suggested that the figures be improved and that ones more appropriate to the results obtained and the sample size be used; for example, violin-type graphs are used with small samples, and I would suggest using box plots with the median and interquartile range.*

Response: We appreciate the constructive comment from this reviewer. We agree. We replaced the small-sample distribution plots with boxplots showing medians and interquartile ranges, overlaid with individual patient-level data. (**Fig 2; Fig 3**)

*Figure 4 should be replaced by a heatmap showing individual variability in the microbiota.*
Response: We appreciate the constructive comment from this reviewer. We replaced Fig 4 with a species-level heatmap showing individual variability in pre- and post-treatment endometrial microbiota profiles for both oral and vaginal supplementation groups. (**Fig 4**)

*Carefully review the manuscript and correct grammatical and typographical errors.*

Response: We appreciate the constructive comment from this reviewer. We carefully reviewed the manuscript and corrected minor typographical and grammatical errors.

*It is important to state that this is a single-center retrospective study.*

Response: We appreciate the constructive comment from this reviewer. We stated that this was a single-center retrospective cohort study in the Abstract and Methods. The revised text is shown in blue in the manuscript. (**Page 2, Line 30; Page 4, Line 83**)

*Congratulations on your work!*

We sincerely appreciate your constructive comments.

Again, we sincerely appreciate your efficient handling of the manuscript and useful comments of the reviewers to improve the quality of the manuscript. We hope that we have improved this manuscript by taking the editor’s and reviewers’ comments into consideration.

Sincerely yours,

Yasushi Hirota, MD/PhD.

Professor

Department of Obstetrics and Gynecology

Graduate School of Medicine,

The University of Tokyo

7-3-1 Hongo, Bunkyo-ku, Tokyo 113-8655 Japan

Phone: +81-3-3815-5411 / Fax: +81-3-3816-2017

E-mail: hirotay-gyn@h.u-tokyo.ac.jp
