# Supplementary material for: Oral versus vaginal Lactobacillus supplementation for endometrial dysbiosis: A paired pre and post–endometrial microbiome study
Source: PLoS One. 2026 Oct 26;21(10):e0360619. doi: 10.1371/journal.pone.0360619 (PMC13704797; doi:10.1371/journal.pone.0360619)
Supplement: Peer review history

# Peer review: Decision Letter 0

**DOI:** 10.1371/journal.pone.0360619.r001

**Authors:** António Machado

21 May 2026

Dear Dr. Hirota,

Thank you for submitting your manuscript to PLOS ONE. After careful consideration, we feel that it has merit but does not fully meet PLOS ONE’s publication criteria as it currently stands. Therefore, we invite you to submit a revised version of the manuscript that addresses the points raised during the review process.

Please submit your revised manuscript by Jul 05 2026 11:59PM. If you will need more time than this to complete your revisions, please reply to this message or contact the journal office at plosone@plos.org. When you're ready to submit your revision, log on to https://www.editorialmanager.com/pone/ and select the 'Submissions Needing Revision' folder to locate your manuscript file.

- A letter that responds to each point raised by the academic editor and reviewer(s). You should upload this letter as a separate file labeled 'Response to Reviewers'.
- A marked-up copy of your manuscript that highlights changes made to the original version. You should upload this as a separate file labeled 'Revised Manuscript with Track Changes'.
- An unmarked version of your revised paper without tracked changes. You should upload this as a separate file labeled 'Manuscript'.

If you would like to make changes to your financial disclosure, please include your updated statement in your cover letter. Guidelines for resubmitting your figure files are available below the reviewer comments at the end of this letter.

If applicable, we recommend that you deposit your laboratory protocols in protocols.io to enhance the reproducibility of your results. Protocols.io assigns your protocol its own identifier (DOI) so that it can be cited independently in the future. For instructions see: https://journals.plos.org/plosone/s/submission-guidelines#loc-laboratory-protocols. Additionally, PLOS ONE offers an option for publishing peer-reviewed Lab Protocol articles, which describe protocols hosted on protocols.io. Read more information on sharing protocols at https://plos.org/protocols?utm_medium=editorial-email&utm_source=authorletters&utm_campaign=protocols.

As the corresponding author, your ORCID iD is verified in the submission system and will appear in the published article. PLOS supports the use of ORCID, and we encourage all coauthors to register for an ORCID iD and use it as well. Please encourage your coauthors to verify their ORCID iD within the submission system before final acceptance, as unverified ORCID iDs will not appear in the published article. *Only*  the individual author can complete the verification step; PLOS staff *cannot*  verify ORCID iDs on behalf of authors.

We look forward to receiving your revised manuscript.

Kind regards,

António Machado

Academic Editor

PLOS One

**Journal Requirements:**

1. When submitting your revision, we need you to address these additional requirements.

Please ensure that your manuscript meets PLOS ONE's style requirements, including those for file naming. The PLOS ONE style templates can be found at

https://journals.plos.org/plosone/s/file?id=wjVg/PLOSOne_formatting_sample_main_body.pdf and

https://journals.plos.org/plosone/s/file?id=ba62/PLOSOne_formatting_sample_title_authors_affiliations.pdf

2. We note that the grant information you provided in the ‘Funding Information’ and ‘Financial Disclosure’ sections do not match.

When you resubmit, please ensure that you provide the correct grant numbers for the awards you received for your study in the ‘Funding Information’ section.

3. Thank you for stating in your Funding Statement:

This research was supported by JSPS (Grant Numbers JP23K15827, JP24K23524, JP23K27176, JP23K24481, JP24K22157, JP23K23803, and JP24K21911), AMED (Grant Numbers JP24gn0110085, JP24gn0110069, JP24gk0210039, and JP24lk0310083), JST (Grant Number JPMJFR210H), and the Children and Families Agency (Grant Number JPMH23DB0101). The funders had no role in study design, data collection and analysis, decision to publish, or preparation of the manuscript.

Please provide an amended statement that declares *all* the funding or sources of support (whether external or internal to your organization) received during this study, as detailed online in our guide for authors at http://journals.plos.org/plosone/s/submit-now.  Please also include the statement “There was no additional external funding received for this study.” in your updated Funding Statement.

Please include your amended Funding Statement within your cover letter. We will change the online submission form on your behalf.

4. Please include captions for your Supporting Information files at the end of your manuscript, and update any in-text citations to match accordingly. Please see our Supporting Information guidelines for more information: http://journals.plos.org/plosone/s/supporting-information.

5. If the reviewer comments include a recommendation to cite specific previously published works, please review and evaluate these publications to determine whether they are relevant and should be cited. There is no requirement to cite these works unless the editor has indicated otherwise.

**Additional Editor Comments:**

Dear authors,

I am pleased to say that the reviewers enjoyed the manuscript very much and we are excited about the possibility of publishing your work. However, the authors need to properly answer all reviewers´concerns and realize the rectifications in the original manuscript. Reviewer 2 recommended rejection and other two reviewers recomended major revisions, reporting several concerns and the need for major revisions. Please read carefully all reviewers’ reports addressing and answering all comments and suggestions, particularly Reviewer 2.

So, I kindly invite the authors to realize a thoughtful revision of the submitted manuscript to achieve publication endorsement by the reviewers.

Thank you and best regards,

António Machado

Reviewers' comments:

**Comments to the Author**

1. Is the manuscript technically sound, and do the data support the conclusions?

Reviewer #1: Partly

Reviewer #2: No

Reviewer #3: Partly

**********

2. Has the statistical analysis been performed appropriately and rigorously?

Reviewer #1: I Don't Know

Reviewer #2: No

Reviewer #3: No

**********

3. Have the authors made all data underlying the findings in their manuscript fully available?

The PLOS Data policy

Reviewer #1: No

Reviewer #2: No

Reviewer #3: Yes

**********

4. Is the manuscript presented in an intelligible fashion and written in standard English?

Reviewer #1: Yes

Reviewer #2: Yes

Reviewer #3: Yes

**********

**Reviewer #1:**  Summary

This manuscript presents a retrospective paired pre–post study comparing oral versus vaginal Lactobacillus supplementation in women with endometrial dysbiosis. The study addresses a clinically relevant question and shows a greater increase in Lactobacillus with vaginal administration. However, important methodological limitations affect the strength of the conclusions.

Major Comments

1. Non-randomized design introduces potential selection bias and confounding.

2. Small sample size (n=32) limits statistical power and robustness.

3. Risk of contamination in low-biomass endometrial microbiome analysis.

4. Lack of species-level microbiome resolution (e.g., L. crispatus vs L. iners).

5. Overinterpretation of exploratory clinical outcomes (live birth rates).

6. Arbitrary threshold of ≥80% Lactobacillus.

7. Use of mean ± SD despite non-normal distributions.

Minor Comments

1. Clarify timing of sampling and menstrual cycle phase.

2. Address antibiotic treatment as potential confounder.

3. Data availability statement does not comply with PLOS requirements.

4. Improve figure clarity and statistical annotation.

5. Minor language refinements needed.

Strengths

- Paired pre–post design

- Same probiotic formulation in both groups

- Clinically relevant topic

- Generally clear writing and transparent discussion

Recommendation

Major Revision

Suggested Closing Statement

This study addresses an important and clinically relevant question using a paired design and standardized intervention. However, significant limitations related to its retrospective, non-randomized design, small sample size, and methodological challenges inherent to low-biomass microbiome analysis limit the strength of the conclusions. I recommend major revision.

**Reviewer #2:**  The authors proposed a retrospective study that compares the effects of oral and vaginal Lactobacillus supplementation in patients with endometrial microbiota dysbiosis who underwent endometrial microbiome testing before and after supplementation. The idea is very attractive but not completely original. Several reports already studied both administration strategies. Authors must improve the manuscript by rewriting it, checking spelling mistakes, and providing more data. In general, the study is interesting but lacks sufficient data to support the main findings.

Comments to authors:

1. Authors need to use the new taxonomic nomenclature for lactic acid bacteria.

2. Line 100: What material is the administered capsule made of? How do you know the capsule material is suitable for oral and vaginal administration? Was bacterial viability assayed? The results of the capsule's resistance to the digestive tract or vaginal environment should be included. A more detailed description of the capsule (manufacturing process, composition, etc.) is necessary. Please include the corresponding information.

3. Line 107: Why were patients receiving antibiotics not excluded?

4. Line 112: It is not indicated when the samples were withdrawn. Was the time for sample collection the same for all patients?

5. Line 127: Sentence is not clear. The results presented in the paper do or do not include patients who received antibiotic treatment.

6. Line 134: Why did the authors choose 10% and 80% as cut-off points?

7. Lines 141-143: It is not clear why the authors performed a statistical study, but they showed data not used for statistics. Please use the same data for both the results and statistical study.

8. Line 163: It is not indicated if assayed lactobacilli are resistant or sensitive to the antibiotics.

9. Lines 163-170: 6/14 patients had less than 90% of the Lactobacillus proportion, and in the oral group, 7/18 patients had 5-90%. Although authors indicate differences are statistically significant, data in the manuscript are different. Authors must present the results according to the statistical studies performed.

10. Line 220: The experimental data are not enough to support this statement.

11. Lines 232-234: Why did the author consider antibiotic treatment not important? Furthermore, within a group, some patients received antibiotic treatment but others did not. In this case, not all patients were in the same conditions and therefore should not be compared to the rest of the patients.

12. Line 234: This statement is not correct because authors did not indicate whether lactobacilli are sensitive or resistant to the antibiotic treatment.

13. Line 237: It is not fully understood how the authors defined "efficiency," and which was the cut-off point?

14. Lines 239-240: The interpretation is biased due to a lack of clarity as previously indicated.

15. Line 254: The results are not enough to support this statement.

16. Line 256: Why do authors refer to subclinical inflammation if it has not been mentioned before as a factor? No inflammatory or immunological parameters were evaluated.

17. Lines 259-261: This sentence highlights the lack of results to support the interpretations.

18. Lines 282-284: The limitations of the study are too significant to ignore them; therefore, the conclusions are not fully supported.

**Reviewer #3:**  The manuscript is significant for its comparison of Lactobacillus supplementation administered orally versus vaginally in patients with endometrial dysbiosis, and it further contributes by providing a before-and-after comparison of treatment outcomes. However, the study has several limitations not mentioned in the text, such as a small sample size, the absence of clear randomization, and its single-center, retrospective design. Additionally, there are potential selection biases, cases of antibiotic use, missing information (primarily in the methodology and results), limitations inherent to 16S rRNA analysis at the genus level, risk of sample contamination, etc. Therefore, major changes are suggested, and the results and conclusions should be formulated with greater caution; the methodology and results should be described in greater detail, and additional analyses should be conducted. Specifically, I suggest the following:

In the abstract, infertility is not linked to the rationale for conducting the study; I recommend improving the wording and also specifying the type of sample used to compare the microbiome

Lines 52–58: The importance of the study is established here, but there are only three references in the entire paragraph. Please provide stronger support for the claims made.

Lines 65–66: Include references

Line 70: The term “dysbiosis” is specific to the microbiota; please change “endometrial microbiota dysbiosis” to “endometrial dysbiosis” and review this throughout the manuscript.

Lines 69–73: The objective is written in several sentences, which makes it confusing; please rewrite it as a single paragraph with an appropriate structure to ensure clarity.

Lines 77–84: Include the date on which the ethics committee approved the project.

Lines 91–92: Explain who linked the information to the records (since it is later explained that it was not the authors).

Provide information on the excluded patients (due to lack of follow-up and pregnancy) because, given the small sample size, pregnancy in particular would be a relevant clinical outcome that could alter the interpretation of results regarding the treatment’s effectiveness. Include this information in the diagram (Figure 1).

Line 94: Indicate whether the pre-treatment microbiome results were obtained using the same sample and technique as the post-treatment results.

Lines 98–100: Explain whether the capsules contained any other excipients, whether there was a specific coating, provide further details on the composition of the capsules, and highlight whether there were any differences between those administered orally and those administered vaginally. It is also necessary to detail the route and dosage of administration for both routes, particularly what recommendations were given for administration. Another important point would be to clarify how the route of administration was chosen for each patient—was it randomized?

It is noted that some patients received antibiotic treatment, and this treatment may have altered the endometrial and vaginal microbiota; therefore, this should be treated as a relevant confounder. A table could be included showing individual patient information to identify who used antibiotics, which route of administration was used for the Lactobacillus supplement, pre- and post-treatment microorganism percentages, and reproductive outcomes.

Lines 112–116: Detail the extraction procedure, sequencing (amplified region, primers, quality results, read depth, controls), and bioinformatic analyses performed

Line 179: “Lactobacillus” should be in italics; please check that this error does not appear elsewhere in the manuscript, including tables and figures

Explain what software was used to create the figures

I recommend avoiding interpreting the lack of significance in Table 1 as due to small sample sizes.

Table 2 contains little data that could be explained in the text; I suggest removing it.

The risk of bias acknowledged by the authors may be a significant limitation and could even directly affect the study’s main conclusion; therefore, statements should be tempered, and it should be clarified what could not be demonstrated.

The statistical analyses should be more robust and should include confidence intervals; it is also important to justify the sample size. Median values or effect estimators with confidence intervals, hazard ratios, or sensitivity differences with adjusted models could be included.

The section on reproductive outcomes should be presented as exploratory analyses because patients with intermediate values are excluded; alternatively, the results for this group could be included to provide more comprehensive analyses indicating what occurred with patients in the intermediate group.

Please avoid any causal inferences throughout the manuscript; carefully review the wording to ensure this. In particular, the conclusions have a causal tone that emphasizes the superiority of vaginal administration. Although the finding is interesting, the study’s limitations do not allow for a definitive conclusion that vaginal administration is superior. Please rephrase the conclusion and similar statements throughout the manuscript.

Indicate whether or not there were any adverse effects associated with the administration of the supplement; also explain whether the capsules’ viability was assessed and whether all necessary authorizations were obtained for both administration routes.

There is a discrepancy regarding data availability, as the authors state that all data are available without restriction; however, some information cannot be shared publicly due to the nature of the study.

It is suggested that the figures be improved and that ones more appropriate to the results obtained and the sample size be used; for example, violin-type graphs are used with small samples, and I would suggest using box plots with the median and interquartile range.

Figure 4 should be replaced by a heatmap showing individual variability in the microbiota.

Carefully review the manuscript and correct grammatical and typographical errors.

It is important to state that this is a single-center retrospective study.

Congratulations on your work!

**********

what does this mean?). If published, this will include your full peer review and any attached files.

If you choose “no”, your identity will remain anonymous but your review may still be made public.

**Do you want your identity to be public for this peer review?**  For information about this choice, including consent withdrawal, please see our Privacy Policy

Reviewer #1: No

Reviewer #2: No

Reviewer #3: **Yes:**  Sandra Pamela Cangui-Panchi

**********

[NOTE: If reviewer comments were submitted as an attachment file, they will be attached to this email and accessible via the submission site. Please log into your account, locate the manuscript record, and check for the action link "View Attachments". If this link does not appear, there are no attachment files.]

To ensure your figures meet our technical requirements, please review our figure guidelines: https://journals.plos.org/plosone/s/figures

You may also use PLOS’s free figure tool, NAAS, to help you prepare publication quality figures: https://journals.plos.org/plosone/s/figures#loc-tools-for-figure-preparation.

NAAS will assess whether your figures meet our technical requirements by comparing each figure against our figure specifications.

# Author comment: Author response to Decision Letter 1

**DOI:** 10.1371/journal.pone.0360619.r002

20 Jun 2026

We sincerely thank the Academic Editor and the reviewers for their careful and constructive comments. We have addressed all comments and substantially revised the manuscript accordingly. A detailed point-by-point response has been uploaded as a separate “Response to Reviewers” file. All changes are indicated in blue font in the revised manuscript.

Attachment Submitted filename: Response to Reviewers.docx

# Peer review: Decision Letter 1

**DOI:** 10.1371/journal.pone.0360619.r003

**Authors:** António Machado

29 Sep 2026

Oral versus vaginal Lactobacillus supplementation for endometrial dysbiosis: a paired pre and post–endometrial microbiome study

PONE-D-26-07839R1

Dear Dr. Hirota,

The revised manuscript was reassessed by one of the two reviewers who had originally recommended major revision, and this reviewer endorsed the revised version. The second reviewer was unavailable to evaluate whether all suggested revisions had been fully addressed. Despite efforts over approximately one month, it was not possible to secure an additional reviewer. To avoid further delay in the editorial process, I therefore carefully evaluated the revised manuscript and the authors’ responses myself.

In particular, I independently reviewed the authors’ point-by-point responses to Reviewer 1 and verified the corresponding amendments in the revised manuscript. In my assessment, all substantive recommendations raised by Reviewer 1 have been adequately addressed. The authors have clarified the non-randomized study design and concomitant treatments, strengthened the methodological description and statistical presentation, acknowledged the limitations associated with the small sample size and potential confounding, addressed the risk of contamination inherent to low-biomass microbiome analysis, added species-level analyses, and appropriately tempered the interpretation of the exploratory reproductive outcomes.

The remaining limitations are inherent to the retrospective study design and are now transparently acknowledged and appropriately discussed in the manuscript. Overall, I consider that the authors have satisfactorily addressed the major concerns raised during peer review and that the revised manuscript has reached the scientific quality required for publication.

Thank you for choosing PLOS ONE.

Best regards,

António Machado

Reviewers' comments:

Reviewer's Responses to Questions

**Comments to the Author**

Reviewer #3: All comments have been addressed

**********

2. Is the manuscript technically sound, and do the data support the conclusions?

Reviewer #3: Yes

**********

3. Has the statistical analysis been performed appropriately and rigorously?

Reviewer #3: Yes

**********

4. Have the authors made all data underlying the findings in their manuscript fully available?

The PLOS Data policy

Reviewer #3: Yes

**********

5. Is the manuscript presented in an intelligible fashion and written in standard English?

Reviewer #3: Yes

**********

Reviewer #3: (No Response)

**********

what does this mean?). If published, this will include your full peer review and any attached files.

If you choose “no”, your identity will remain anonymous but your review may still be made public.

**Do you want your identity to be public for this peer review?**  For information about this choice, including consent withdrawal, please see our Privacy Policy

Reviewer #3: **Yes:**  Sandra Pamela Cangui Panchi

**********

# Editor report: Acceptance letter

**DOI:** 10.1371/journal.pone.0360619.r004

**Authors:** António Machado

PONE-D-26-07839R1

PLOS One

Dear Dr. Hirota,

I'm pleased to inform you that your manuscript has been deemed suitable for publication in PLOS One. Congratulations! Your manuscript is now being handed over to our production team.

At this stage, our production department will prepare your paper for publication. This includes ensuring the following:

* All references, tables, and figures are properly cited

* All relevant supporting information is included in the manuscript submission,

* There are no issues that prevent the paper from being properly typeset

You will receive further instructions from the production team, including instructions on how to review your proof when it is ready. Please keep in mind that we are working through a large volume of accepted articles, so please give us a few days to review your paper and let you know the next and final steps.

Lastly, if your institution or institutions have a press office, please let them know about your upcoming paper now to help maximize its impact. If they'll be preparing press materials, please inform our press team within the next 48 hours. Your manuscript will remain under strict press embargo until 2 pm Eastern Time on the date of publication. For more information, please contact onepress@plos.org.

You will receive an invoice from PLOS for your publication fee after your manuscript has reached the completed accept phase. If you receive an email requesting payment before acceptance or for any other service, this may be a phishing scheme. Learn how to identify phishing emails and protect your accounts at https://explore.plos.org/phishing.

If we can help with anything else, please email us at customercare@plos.org.

Thank you for submitting your work to PLOS One and supporting open access.

Kind regards,

PLOS One Editorial Office Staff

on behalf of

Dr. António Machado

Academic Editor

PLOS One
